# A Systematic Review of Global Health Assessment for Education in Healthcare Professions

**DOI:** 10.5334/aogh.3389

**Published:** 2022-01-06

**Authors:** Connor Sharon E., Jonkman Lauren J., Covvey Jordan R., Kahaleh Abby A., Park Sharon K., Ryan Melody, Klein-Fedyshin Michele, Golchin Negar, Veillard Regine Beliard

**Affiliations:** 1 University of Pittsburgh School of Pharmacy, Pittsburgh, Pennsylvania, US; 2 Duquesne University School of Pharmacy, Pittsburgh, Pennsylvania, US; 3 Roosevelt University College of Science, Health, & Pharmacy, Chicago, Illinois, US; 4 Notre Dame of Maryland University, Baltimore, Maryland, US; 5 University of Kentucky College of Pharmacy, Lexington, Kentucky, US; 6 University of Pittsburgh Health Sciences Library System, Pittsburgh, Pennsylvania, US; 7 University of Washington, Seattle, WA, US; 8 GlaxoSmithKline US Medical Affairs, Baltimore, MD, US

## Abstract

**Objective:**

Emphasis on global health education is growing, with schools/colleges developing relevant courses, areas of concentration, and other didactic content. Organizations such as the Consortium of Universities for Global Health (CUGH) provide guidance for competencies in global health, but evaluation strategies are lacking. Accordingly, the purpose of this study was to identify methods and tools utilized to assess knowledge, skills, and attitudes in global health courses for health science students.

**Methods:**

A systematic review was conducted according to the PRISMA guidelines. The initial search was conducted using controlled vocabularies to screen PubMed, EMBASE, Global Health using Ovid, CINAHL, and ERIC from January 1997 to March 2020. Included articles detailed students in health professions, described a didactic educational intervention related to global health, and described assessment strategies and results.

**Results:**

A total of 12,113 titles/abstracts were identified. Based on the study inclusion criteria, 545 full texts were reviewed, and 79 full-text articles were selected for qualitative synthesis. Findings of the research revealed that cultural competence (70.9%) was evaluated most often, followed by health disparities (26.6%) and global health itself (12.7%). Most articles used quantitative assessment methods (86.1%), with surveys being the predominant method. A total of 91.1% of studies assessed perceptions, attitudes, and beliefs, while fewer evaluated knowledge (43.0%) and skills (19.0%). The most common validated tool employed was the Inventory for Assessing the Process of Cultural Competence (IAPCC).

**Conclusions:**

Based of the results of this study, the majority of the assessment tools utilized for global health education focused on cultural competence. One of the important findings of this research is the lack of validated instruments to assess students’ skills in health disparities and global health. Given the recent global pandemic, these skills are essential for educating health care professionals to enhance global health.

## Introduction

Although there are various definitions of global health, one that has become commonly accepted is “an area for study, research, and practice that places a priority on improving health and achieving health equity for all people worldwide [1].” The demand for global health education within health sciences has grown considerably over the last several decades [2,3]. Students are eager to participate in these experiences for a variety of reasons, including development of cultural competency, desire to reduce health disparities, interest in various cultures, and enhancement of their global health competencies prior to graduation [4].

There is a plethora of models that exist for global health education, ranging from didactic coursework to experiential opportunities abroad. While there has been considerable focus in the literature on experiential education, there have been fewer publications on the didactic approaches. Arguably, this latter format may have potential for the most impact. Given the considerable financial burden of higher education, many students are unable to travel and engage in immersion, For the students who do take advantage of opportunities abroad, baseline didactic engagement in global health should be viewed as a prerequisite, either separately delivered or as preparation prior to an international experience.

An additional challenge to furthering knowledge in this area is the siloed nature of higher education, and accordingly, the scholarly assessments associated with global health. Studies describing global education initiatives tend to focus on a single healthcare profession and often are published in associated professional journals. The field of global health work is interprofessional, as are healthcare teams. This highlights the need for more inclusivity. While some fledgling work has been done to describe interprofessional global health education [5,6,7,8,9], there is still a gap in the literature and few venues for publishing such work.

Several organizations and programs have aimed to standardize educational experiences through the establishment of formal competencies [10,11,12]. Jogerst et al. described an effort through the Consortium of Universities of Global Health (CUGH) to establish a list of interprofessional global health competencies [11]. A finalized list composed of 13 competencies across eight domains at the “Global Citizen” level (those for post-secondary students pursuing any field with bearing on global health) has been developed. In addition, 38 competencies across 11 domains for the “Basic Operational Program-Oriented” level (those required for students aiming to spend a moderate amount of time working in global health) have been published. Despite standardization of these competencies, there is no method for assessment of students’ performance to meet these competencies. In fact, Jogerst et al. specifically identified the need for more assessment tools in the area of global health education [11]. Additionally, Eichbaum suggests that lacking are comprehensive global health assessments to ensure learners are equipped with a full understanding of the factors that affect patient outcomes [13].

Given these findings in the literature, the purpose of this systematic review was to identify methods and tools utilized to assess knowledge, skills, and attitudes in global health courses for health science students.

## Methods

### General description

This research is a collaborative effort between faculty members at the American Association of Colleges of Pharmacy (AACP) and a clinical health sciences librarian. The systematic literature review was conducted and reported according to the Preferred Reporting Items for Systematic Reviews and Meta-Analysis (PRISMA) guidelines [14]. A search was performed to obtain all relevant publications regarding didactic global health education assessment within a ten-year timeframe among a selected set of diverse healthcare professional schools. The research protocol was written and registered in the International Prospective Register of Systematic Reviews (PROSPERO) [15]. The review process consisted of the following four phases: (1) an initial search to identify articles exemplifying the desired retrieval, (2) two sets of title/abstract reviews of the entire search results, (3) full-text review, and (4) narrowing the results to those articles that met the study objective.

### Search/review strategy

A pilot search was conducted to identify target global health assessment strategies to develop the creation and validation of the formal search strategy. Search vocabulary was then composed to comprehensively search the concepts of global health and educational assessment tools by including concepts related to global health, cultural competence, education methods, assessment techniques, and evaluative tools and techniques for educational assessment. Boolean operators combined these concepts into the search strategy composed by the clinical health sciences librarian. Exclusion terminology eliminated education articles related to specific global health disease burdens (e.g., smoking). The librarian designed the original search in PubMed (Appendix A) and returned it to committee for input; after review and revision, the strategy was translated using the controlled vocabularies and syntax to other databases, including EMBASE, Global Health using Ovid, and CINAHL and ERIC via EBSCO. The initial searches covered January 1, 1997, to September 21, 2017; a search update was also conducted from the end of the initial search through to March 2020. This search end date was mutually agreed upon by the research team to avoid the impact of the COVID-19 pandemic, which has radically impacted global health education. The team reviewed references from included articles for relevant additional publications.

Search results were exported to EndNote (Clarivate Analytics; Philadelphia, PA). DistillerSR (Evidence Partners Incorporated; Ottawa, Canada), a web-based application for conducting systematic reviews, was used to conduct the remaining phases of the review [16]. The review team consisted of ten pharmacy researchers/educators with experience/knowledge in global health education, and several members of the team with previous experience conducting systematic reviews. For the first title/abstract review, each record was reviewed by two individuals independently, with conflicts automatically moved forward. For the second title/abstract review, conflicts between independent reviewers were resolved by a third party. For the full-text review, records were again reviewed by two independent researchers, with conflicts resolved by group discussion and consensus.

### Inclusion/exclusion criteria

Articles were screened based on inclusion/exclusion criteria. The full-text articles that were included in this study described didactic education and associated assessment techniques to teach global health in US-based settings to healthcare professional students.

For the purpose of the study, “didactic education” was defined as education delivered live or online in a formal educational environment in the United States, inclusive of higher education classroom training and service-learning opportunities. “Global health” included educational efforts specific to the term itself, as well as related efforts focused on health disparities (health difference that is closely linked with social, economic, and/or environmental disadvantage [17]). Additional global health related terms included cultural competency (behaviors, attitudes, and policies that come together in a system or agency, or among professionals, and enable that system or agency or those professions to work effectively in cross-cultural situations [18]). “Healthcare professional students” were defined as students in programs for medicine (including osteopathy and physician assistants), dentistry, nursing (including midwifery), and pharmacy [19]. “Assessment” included strategies used to measure learning, either focused on knowledge, skills, or attitudes/perceptions/beliefs related to the topic areas of interest; these strategies were inclusive of standard tools (validated or not) as well as other informal methods/analysis.

General exclusion criteria included the following: non-English articles and non-peer reviewed publications (case studies, editorials, reviews, commentaries, thesis, and summaries). Specific exclusions were articles that described experiential educational opportunities (without didactic components), those related to education based at non-US institutions, community-based efforts not centered within higher education, and analyses lacking quality descriptions of assessment methods and results. This last requirement expected that educational strategies were evaluated in a process that declared a formalized research objective (i.e., not merely a descriptive paper), adequately described the methodology and assessment, and that results of the evaluation were fully detailed. For instance, qualitative studies providing only summary and exemplar quotes from learners were not included in this systematic review, nor were generic course evaluations.

### Extraction

The articles selected for full-text review were compared with the initial set of results identified as exemplary for literature review, as a quality check for the process implemented. A qualitative synthesis of the final included articles was performed by extracting relevant data, organized into the extraction table, including author name/year, study objectives, health professional students involved, setting, sample (size, inclusion and exclusion criteria if present, demographics), assessment method utilized, and quantitative/qualitative results. A standard process for quality evaluation of each article was not possible due to the significant diversity in methodology across the final result set, but the limitations associated with each article were identified. Assessment methods within included articles (of which overlap was possible) were categorized by: (1) topic area (global health, health disparities, and cultural competency), (2) outcome assessed (knowledge, skills, and attitudes/perceptions/beliefs), (3) qualitative versus quantitative methods utilized, and (4) whether the method was validated based on available information in each manuscript.

## Results

### Overall study results

**Figure 1:**
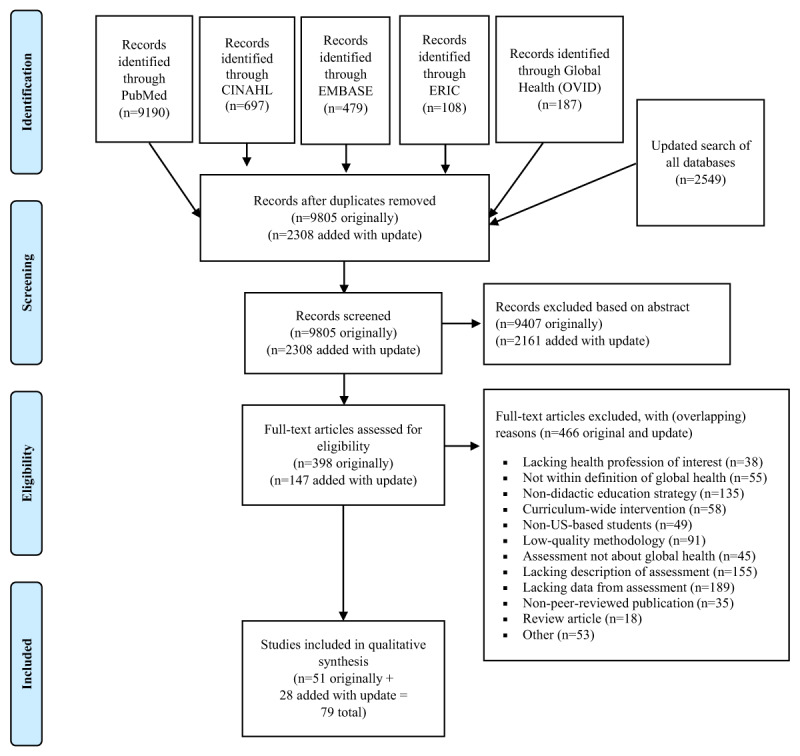
Adapted PRISMA Flow Diagram (2009).

The PRISMA flow chart for the systematic review is shown in ***Figure 1***. A total of 9805 titles/abstracts were identified by the original search and 2308 in the updated search. Of these, 398 full texts from the original search and 147 full texts from the updated search (final total of 545) were reviewed for inclusion. A final set of 51 full-text articles in the primary search [20,21,22,23,24,25,26,27,28,29,30,31,32,33,34,35,36,37,38,39,40,41,42,43,44,45,46,47,48,49,50,51,52,53,54,55,56,57,58,59,60,61,62,63,64,65,66,67,68,69,70], as well as 28 full-text articles in the updated search [71,72,73,74,75,76,77,78,79,80,81,82,83,84,85,86,87,88,89,90,91,92,93,94,95,96,97,98] were selected for qualitative synthesis. Summarized details for each study are included in ***Table 1***.

**Table 1:** Description of the Included Studies.


AUTHOR NAME (YEAR)	STUDY OBJECTIVE(S)	SAMPLE SIZE AND DESCRIPTION OF PARTICIPANTS	DESCRIPTION OF SETTING	ASSESSMENT METHOD UTILIZED	STUDY RESULTS	STUDY LIMITATIONS

**Addo-Atuahet al. (2014) [20]**	Describe a global health elective course within a PharmD curriculum designed to provide pharmacy students with the requisite skills and knowledge to help them pursue global health careers in the public health arena and to examine students’ perceptions of the instructional techniques used and the skills set developed during the course	100 65% women; 58% born outside USA; 88% travelled outside USA	Touro College of Pharmacy, “*Global Health*” 15-week, three-credit semester blended (two-hour classroom and one-hour online) elective course	Investigator-designed 19-item pre-post analysis on knowledge/attitudes (three items), role perception/career opportunities (two items), instructional techniques used (six items) and skill development (eight items) using a five-point Likert scale. Open-ended items for overall impression, experiences, and recommendations.	Significant improvements from pre- to post-survey were seen for the skill development domain (*p* = 0.0001) but not for knowledge and attitudes (*p* = 0.14), role perception and career opportunities (*p* = 0.21), or instructional techniques used (*p* = 0.14), although numerical improvements were seen.	Lack of formal qualitative data analysis

**Alexander-Ruff and Kinion (2018) [82]**	Examine undergraduate student nurse perceptions of cultural consciousness following a one-week, seven consecutive days, CISL experience in a rural American Indian Reservation Community	30 90% Caucasian, 6.7% American Indian, 3.3% Asian; 46.7% accelerated degree students, 53.3% traditional bachelor’s degree	Montana State University and Northern Plains Indian Reservation seven-day CISL with pre-immersion activities	Qualitative data collected from (1) instructor/preceptor observations during CISL; (2) student reflections immediately after the CISL experience; (3) follow-up student reflections approximately 2–3 weeks after the CISL.	Student stated goals surrounded three themes: skill development, make a difference, and cultural engagement. 76.7% of students voiced feelings of despair due to the lack of control over the environment the community faces.	Evidence of sustained cultural consciousness over time; lack of faculty involvement without ongoing practice in a non-dominant cultural environment

**Allenet al. (2013) [21]**	Add to the understanding of the role of international healthcare experiences on self-perceived cultural competency among health professions students who participated in a three-week community-focused immersion course in Peru	77 89.6% women; mean age 24.4 years; 84% nursing, 14% pharmacy, 1% nutrition; 100% no previous experience in Peru; 13% somewhat fluent in Spanish; 12% never been to another country, 9% born in another country; 35% limited to no contact with cultures other than own	Washington State University, “*Health Care in Peru*” program Three-credit interdisciplinary experience open to any health professions student, including five pre-seminars (two hours per seminar) and three weeks in Peru	28-item CCCHS [105] assessing self-comfort with skills (11 items), perceived knowledge (eight items) and self-awareness (five items) with five-point Likert scales. Four additional items were added for other abstract concepts.	Overall cultural competency increased from 90.4 ± 15.3 to 107.9 ± 15.3. Knowledge increased from pre- to post-surveys (20.9 ± 5.5 to 26.7 ± 5.9, *p* < 0.001) with significant increases seen on all eight items. Comfort also increased for skills (29.6 ± 6.8 to 45.3 ± 5.9, *p* < 0.001) with increases on 10 of 11 items. Awareness increased (15.7 ± 3.6 to 19.4 ± 3.6, *p* < 0.001). Improvements were seen on all four abstract concept items as well (*p* < 0.05).	Self-report; failure to measure actual behaviors; limited diversity

**Arif et al. (2017) [22]**	Develop, implement, and assess whether simulated patient case videos improve students’ understanding of and attitudes toward cross-cultural communication in health care	159 66% women, 48.4% Asian American, 39.6% Caucasian, 4.4% African American/Black, 3.1% Hispanic; mean age 25.3 years; 67.6% multilingual, 27% lived outside of US	Midwestern University Chicago College of Pharmacy, workshop in a required healthcare communications course	Pre- and post-survey on cultural self-awareness (adapted from [133,134]). Likert-type assessment during using the SOLER and LEARN models of effective cross-cultural communication. Pre- and post-knowledge assessments.	Knowledge increased from pre- to post- (5.5 vs. 6.6 of 9, *p* < 0.05). Greatest improvement related to appropriate use of interpreters and awareness of verbal models of cross-cultural communication in practice. Student attitudes and comfort with providing cross-cultural care increased after the workshop (*p* = 0.002). Evaluations were positive forward the videos, remarking on perceptions of knowledge gain. 88.7% felt more communication training was needed in the pharmacy curriculum overall.	Single school; small sample size; make-up of student body; outside influences upon results

**Arif et al. (2019) [95]**	Design and implement an elective course that prepares student pharmacists to provide culturally sensitive health care by developing their knowledge, self-confidence, and clinical and communication skills for working with patients from various cultural backgrounds during community health screenings	62 (31 intervention and 31 control) 37.5% intervention and 22.6% control self-reported as white; 65.6% in either group were women; mean age 25.4 years in intervention and 26 years in control; 48.4% second-year and 51.6% third-year in either group	Midwestern University Chicago College of Pharmacy, *“Health Promotion and Disease Prevention Across* *Cultures”* One-credit hour elective course for second- and third-year students over six weeks	Investigator-designed pre- and post-survey including demographics, a 12-item knowledge quiz on disease state knowledge, and 13 self-assessment items on perceived confidence in knowledge and skills.	Post-survey knowledge assessment scores among students in the course were numerically higher than controls (74.2% vs 68.3%, respectively), but not significantly (p = 0.07). However, there was a significant increase in knowledge overall (pre-course 66.4% vs post-course 74.2% correct, p = 0.03). Students in the course had increased confidence for knowledge of other beliefs and cultural health practices, more appreciation for the importance of providing culturally competent care and inquiring about a patient’s cultural background than did the control group (p < 0.05).	Single school; small sample size; make-up of student body; rigor of knowledge questions; translation of confidence to practice

**Assemi et al. (2004) [23]**	Implement and assess the impact of a cultural competency training course designed specifically for pharmacy students	56 74% women; 48% first-year, 45% second-year, 3% third-year and 3% fourth-year	University of California San Francisco College of Pharmacy, “*What is Cultural Competency in Pharmaceutical Care?*” One-day (eight-hour) elective based in part on training and curricular materials available from The Network in the Center for the Health Professions at UCSF	Investigator-designed pre-post 12-item survey on perceptions of awareness, knowledge, and communication skills using a five-point Likert scale.	All items increased significantly from pre- to post-training (*p* < 0.001). Change in total score did not differ significantly between the students in the first vs second course offering (14.04 vs 15.01, respectively; *p* > 0.05).	Lack of validation; translation of confidence to practice; small sample, generalizability; confounding from student sociodemographic characteristics or prior training in cultural competency

**Assemi et al. (2006) [24]**	Examine the psychometric properties of a 12-item scale designed to estimate the impact of cultural competence training on pharmacy students’ self-efficacy in providing culturally appropriate patient care	173 71% women; 45% first-year students, 35% second-year, 18% third-year, 1% fourth-year	University of California San Francisco College of Pharmacy, “*What is Cultural Competency in Pharmaceutical Care?*” One-day (eight-hour) elective based in part on training and curricular materials available from The Network in the Center for the Health Professions at UCSF	Investigator-designed pre-post 12-item survey on perceptions of awareness, knowledge, and communication skills using a five-point Likert scale.	Post-survey scores were significantly higher than pre-survey scores (post- 47.96 ± 5.15, range 36–60 vs pre- 34.21 ± 6.19, range 15–53; *p* < 0.001).	Self-report; ability to differentiate; changes in scale responses over time

**Boyer et al. (2020) [87]**	Increase racial/ethnic and cultural competency with diverse patients among PMHNPs through (1) coursework emphasizing the role of race/ethnicity and culture in the presentations and perceptions of behavioral health and (2) the impact of the intervention on clinical behaviors over time.	26 88.5% women; mean age 39 years; 76.9% non-Hispanic white, 7.7% Hispanic, 3.9% Asian American, 7.7% Black/African American, and 3.9% American Indian	University of Colorado Denver College of Nursing 15-week course on integrated behavioral healthcare, including modules on culturally competent care	Pre- and post-assessment using the 24-item ESI [115] self-reported their racial/ethnic sensitivity when working with patients.	At course conclusion, students reported higher perceived sensitivity (4.29 ± 0.36) than before (3.92 ± 0.44, p = 0.002). Improvements from pre- to post-course were significant for non-Hispanic white students, but not for racial/ethnic minority students.	Small sample size; lack of a defined control group; single school

**Braun et al. (2017) [26]**	Describe the creation and implementation of a novel student-organized, interprofessional elective to address the LGBTQI educational gap within our institution	273 68% female sex, 66% female gender, 5% transgender, 56% heterosexual; 48% white, 38% Asian, 14% Hispanic; 14% medicine, 9% dentistry, 47% pharmacy, 13% nursing, 12% general public	University of California San Francisco, LGBTQI Health Forum (“*the Forum*”) One-unit 10-contact hour elective	Investigator-created survey to examine participant beliefs and confidence surrounding LGBTQI healthcare using Likert-type scales (one = strongly disagree, six = strongly agree).	Participants indicated a relative lack of LGBTQI health-related content in their core curricula (2.98 ± 1.33). Significant improvements were seen in comfort interacting with LGBTQI patients (*p* < 0.01), preparedness in providing healthcare for LGBTQI patients (*p* < 0.01), knowing where to find more information (*p* < 0.01), listing unique needs of LGBTQI patients (p < 0.01), conducting accurate sexual histories of LGBTQI patients (*p* < 0.01) and student confidence in conducting an accurate and inclusive medical history with an LGBTQI patient (*p* < 0.01).	Only preliminary informal assessment data available; data does not appear to be paired

**Braun et al. (2017) [25]**	Evaluate the impact of an elective course for health profession students in transgender health that was implemented to address these gaps in provider knowledge	46 80% female sex, 76% female gender; 57% white, 24% Asian; 48% pharmacy, 24% medicine, 17% nursing; 78% first year in program	University of California San Francisco, “*Transgender Health”* 10-session, lunch-hour course	Pre-, post-, and three-month post-questionnaires using nine-item transphobia scale (from [116]), investigator questions on knowledge, cultural awareness, disparities and health policy	Knowledge increased (*p* < 0.01) on multiple knowledge subject areas, including terminology (4.67 to 5.21 of 6), primary care (3.39 to 4.85 of 6), medications (3.78 to 4.65 of 8) and federal policy (1.04 to 1.37 of 4). No difference seen for cultural competency and history, California policies or health disparities immediately post-course. Transphobia decreased post-course (15.9 to 14.1 of 45, *p* = 0.0021). For the three-month follow-up, there were increases in knowledge from the immediate post-survey for cultural competency, state policy and federal policy, and no change in transphobia.	Lack of longer follow-up; presence of unmeasured variables; resource/funding/experience limitations; lack of adequate power for stratifications

**Brown et al. (2007) [27]**	Evaluate the effectiveness and impact of an elective service-learning course offered in cooperation with a charitable pharmacy providing services to the surrounding community	33 wNo demographics provided	University of Cincinnati, “*Longitudinal Patient Care*” One- to three-credit elective course	23-item pre-survey and 32-item post-survey (designed by the Center for Healthy Communities, Wright State University (from [83]). Pre-survey on demographics, work history, volunteer experience, 15 items on civic, cultural and social issues, and eight items on post-graduate work placement (using seven-point Likert scales). Post-survey on same 23 items, plus nine items on broader community issues (five-point Likert scale). Reflective writings were also analyzed.	Significant differences were found between pre-and post-survey responses to six of 23 statements (one civic issue, three cultural issues, one health disparities and one post-graduate employment). Ratings of perceived knowledge on all nine items post-survey improved from baseline.	Limited to no analysis of reflective qualitative data; biased sample of students engaged with the topic; subjectivity of the assessment

**Brown et al. (2008) [28]**	Promote the (1) development of interprofessional team behaviors with a secondary goal of (2) fostering the development of cultural competency within health professions students	81 86.4% women; 82.7% Caucasian, 17.3% other; 40.7% ≥24 years; 66.6% undergraduate, 33.3% graduate; 22.2% pharmacy, 32.1% nursing, 18.5% social work, 27.1% allied health;	University of Cincinnati Three-hour quarter-long elective course that focused on providing students opportunity for IPE and cultural competence training	20-item SAQ modified from a similar questionnaire (from [135,136]) that contains 11 items on IPE and nine items on feelings about self and peer assessments. 25-item IAPCC-R (from [137]) on culture.	Improvements were seen for 8/11 IPE items and seven of nine assessment items from pre- to post- (*p* < 0.05) on the SAQ. For the IAPCC-R, improvements were also seen pre- to post- (68.5 ± 7.3 to 75.8 ± 8.69, *p* < 0.001), where students moved from culturally aware to culturally competent.	One-group design; confounding variables; subjective nature of the SAQ; social desirability bias

**Carabez et al. (2015) [29]**	Describe the changes in student knowledge from the beginning to the end of the semester through a description of the assignment and an analysis of the pre-interview and post-interview surveys	112 Age range: 30% less than 26 years, 49% 36–35 years, 21% older than 35 years; 40% unprepared to care for LGBT; > 10% religious conflicts with LGBT care	San Francisco State University, Community/Public Health Nursing course (BSN, MSN, RN to BSN, generic MS)	Investigator-designed 14-item pre-survey (demographics, Likert-type items about perceived knowledge, level of education and comfort, personal impacts on care) and 18-item post-survey (intervention evaluation, perceived knowledge).	Perceived knowledge about sexual orientation and gender identity increased from pre- to post-. Change in knowledge was more for gender identity (t = 19.3, *p* < 0.0001) than for sexual orientation (t = 4.14, *p* < 0.005). 74% reported increased awareness as the result of the intervention. Comments included developing consciousness about the need for education, the value of conducting original research, and becoming aware of unconscious biases.	Limited numerical results available, mainly descriptive results

**Carpenter et al. (2015) [30]**	Compare a web-based curriculum to a traditional lecture format on medical students’ cultural competency attitudes using a standardized instrument and to examine the internal consistency of the standardized instrument	174 (86 experimental and 88 control) 60% men; 77% white or Caucasian, 13.2% Asian or Asian-Indian, 5.7% Black or African American	University of Alabama at Birmingham, “*Introduction to Clinical Medicine*” Required course for first year students randomized to two groups: (1) web-based curriculum (Cultural Competence Online for Medical Practice), or (2) single lecture, focusing on clear communication, overcoming language barriers and eliciting the patient perspective.	15-item HBAS (from [107,108]) to assess cultural competency attitudes, completed within two weeks after training using a six-point Likert scale	No differences were observed in the overall median scores between the intervention (median 5.2; interquartile range 4.9–5.5) and the control group (median 5.3; interquartile range 4.9–5.6, *p* = 0.77). No differences in the median scores was observed between groups for any of the sub-scores.	Single center; short-term outcome, group contamination; lack of assessment of skills, knowledge or patient outcomes

**Chang et al. (2019) [90]**	Assess both the perceived long-term and short-term impact of the one-week service-learning trip in Nogales, Arizona	55 (7 from 2016 trip and 48 former trip participants) No demographics provided	Mount Sinai Icahn School of Medicine Nogales, AZ, trip One-week domestic global health service-learning experience with a preparatory curriculum	Qualitative analysis of journal entries (during the 2016 trip) as well as an investigator-designed 28-item survey to past participants, including 25 items Likert scale items on agreement to learned themes and three open-ended items about strengths, weaknesses, improvement of the experience.	Journals gave seven themes grouped into two categories: explicit (personal benefits; influence on future career; gratitude for exposure) and implicit (shaped view of privileges; inspiration for continued reflection; perceived impact on community; impact of motivations on another’s action) insights and lessons. Students agreed that the experience enhanced their knowledge regarding rural health (100%), medical education (97.9%), awareness of own privilege (97.9%), prevalence of socioeconomic privilege (93.8%), the importance of local community (93.8%) and other attributes.	Sample size; generalizability; response rate to survey; potential for recall bias; lack of control group

**Cox et al. (2014)[31]**	Describe an interprofessional course designed for Doctor of Pharmacy and Master of Social Work students to enhance knowledge and identify barriers in providing care to medically underserved populations with an emphasis on homeless populations and examine students’ perceptions of interprofessional education	18 83% pharmacy; 100% women; 72% prior experience working with medically underserved (children, homeless shelters, free clinics and health fairs, local jail)	Virginia Commonwealth University and a Federally Qualified Health Care for the Homeless Clinic, “*Medical Access and Care for Underserved Populations*” Interprofessional elective graduate course for third-year pharmacy and second-year social work students	Investigator-designed questionnaire using five-point Likert scale, including 10 items on knowledge of medically underserved populations, five items on agreement to statements on medically underserved populations, 11 items on perceptions, and five items on barriers to health services. Student confidence, interest and opinions on interdisciplinary education were also assessed.	Overall knowledge score significant increased from 6.9 ± 2.6 to 8.3 ± 2.1 (*p* = 0.01) in pre to post. Awareness about barriers (e.g., competing needs, transportation issues, communicating with healthcare professionals) were high pre-test and remained the same at the post-test. Students had accurate perceptions about the misconception and truth statements regarding homeless, mentally ill, and racial/ethnic minority populations with few changes from the pre- to post-tests. Pre- scores for comfort and confidence in working with homeless, mentally ill, and various racial groups were high and they tended to increase post-, with a significant increase (*p* = 0.03) in confidence in communicating with different types of underserved populations. Students’ interest in working with underserved populations decreased (*p* = 0.008) from pre to post.	Small sample size; selection and non-response bias; baseline perceptions and high level of knowledge may limit generalizability

**Crandall et al. (2003) [32]**	Summarize the current practice of cultural competency training within medical education and describe the design, implementation, and evaluation of a theoretically based, year-long cultural competency training course for second-year students at Wake Forest University School of Medicine	12 42% men; 58% minority	Wake Forest University School of Medicine, “*Culture and Diversity*” Year-long elective course for second year students to experiment with content and have the students help to identify experiences to be integrated into the four-year curriculum	Pre- and post-assessment using 16-item MAQ (from [104])	Cronbach alpha were 0.88 (pre-course) and 0.89 (post-course). Total scale score increased from 2.31 to 4.02 (*p* < 0.001, Cohen’s D: 3.1) from pre- to post-assessment. Sections for knowledge (2.07 to 3.92, *p* < 0.001, Cohen’s D: 3.5), skills (2.15 to 4.01, *p* < 0.001, Cohen’s D: 2.5) and attitude (2.92 to 4.19, *p* < 0.001, Cohen’s D: 2.2) similarly increased.	Small sample size; selection bias; self-reported data; multi-faceted nature of knowledge, skills may not be related to intervention

**Crocker et al. (2018) [79]**	Examine knowledge, attitudes, and self-reported skills differences among medical students, physicians, and other professionals in the online cultural competency education modules	1745 38% medical students, 17% physicians, and 45% other; 39% from Alabama; 52% from educational institutions	University of Alabama at Birmingham School of Medicine, “*Introduction of Clinical Medicine”* First-year course with four online modules on cultural competence addressing bias and stereotypes from the provider perspective and diet and religion from the patient perspective; modules also were available for CME credit	Investigator-designed module-embedded questions, including 17 items in the four modules (bias, stereotype, diet, religion) addressing knowledge, attitudes, and self-reported skills.	Knowledge scores found that students defined stereotype correctly 84% of the time, compared to 47% for bias. A total of 69% identified that bias impacts decision-making. For attitudes, 98% were able to identify stereotyping in a given scenario, and 99% were able to identify effects upon communication related to bias. A total of 13% identified asking about religious beliefs during typical encounters, but 78% felt like these beliefs impacted reactions to illnesses often.	Unknown if questions represent baseline knowledge or perspectives after modules, tool was not validated; lack of participant information

**Curtin, et al (2014) [33]**	Examine the impact of an international service learning experience using a quantitative and qualitative approach	11 100% Caucasian; age range 21–24 years; 64% seniors; 80% with previous international travel; 73% minimal to some Spanish language skills	University of Rhode Island Three-credit course inclusive of institutional and program mission and goals, global health core content, 11 hours of pre-experience, two weeks on-site, and three post-experience	IES (from [120]) for quantitative data using seven-point Likert scale, ranging from one (low/small) to seven (high/large); content analysis of CRI narratives for qualitative data. Most data were collected post-experience.	Students reported high overall impact (mean = 5.9), and high means for the Professional student nurse role (mean = 6.10 ± 0.74), personal development (mean = 6.08 ± 0.76), international perspectives (mean = 6.03 ±0.71). CRIs provided specific evidence of short-term impacts of the program on careers and effectiveness/efficiency as professional student nurses.	Small sample size; inflation of scores due to administration directly after experience; lack of formal qualitative method for assessment of CRI

**De Oliveira et al. (2015) [34]**	Describe a sequence of scaffolded cross-cultural experiences within a decentralized model of IPE education for physician assistant and physical therapy students that includes a cultural component for applied practice in a diverse society and includes the evaluation of students’ engagement and attitudes towards IPE	71 28% PA, 72% PT	Carroll University Three part decentralized scaffolded IPE exploration series over 12 months	10-item IESS (citation not provided) using four-point Likert scale at end of educational series	100% agreed/strongly agreed with six of 10 items (beneficial to self-growth, importance of interprofessional collaboration, cultural awareness for future practice, greater depth of appreciation for diverse needs, capable of utilizing interpreters and beneficial – should continue). 3.9 to 7.8% disagreed with four of 10 items (unique need met in program, better understanding of other discipline, prepared for interprofessional environment, enjoyed experience). Qualitative feedback focused on exploration of self, examination of various dimensions of culture, and exploration of the intersection between health and culture.	Lack of formal comparison between PT and PA; lack of pre-assessment

**De Bonis (2015) [35]**	Evaluate the impact of service-learning on graduate nursing students’ cultural competence, civic engagement, and knowledge and understanding of the effects of poverty on health care	152 No demographics provided	Allen College (Waterloo, Iowa), “*Advanced Health Assessment*” 16–20 hours of service at Salvation Army for underserved and a service-learning opportunity for graduate nursing students	Investigator-created pre- and post-semester 15-item attitudinal survey using seven-point Likert scale, covering civic engagement, cultural competence, social issues, and health disparities. Post-test also with eight-item knowledge perception rating pre- and post-service (from [83]).	All four items on civic engagement improved after the experience (*p* < 0.001), while three of six items on cultural competence and three of three items on social justice and health disparities improved. For perception of knowledge, ratings on all eight statements improved (*p* = 0.0001).	Lack of a control group; convenience sample; lack of diversity

**Devraj et al. (2010) [36]**	Implement active-learning exercises in a required pharmacy course and assess their impact on students’ knowledge and confidence in identifying and communicating with patients with low health literacy, as part of a required course in cultural competency, health literacy, and health beliefs	76 62% women; 79% white; mean age 22.8 years; 33% prior bachelor’s degrees; 51.3% from southern Illinois	Southern Illinois University, “*Health Promotion and Literacy*” three-credit required course for third-year students	Investigator-designed 20-item pre-, and post-survey instrument to assess knowledge about health literacy, and five items (assessed pre-, retrospective pre- and post-) regarding confidence in the ability to identify, communicate, and assess health literacy. Course assessment at the end of the semester asked about various clusters (cultural competency, health literacy, health beliefs, etc.).	Score on knowledge survey increased from 77.57 to 88.56 from pre- to post-, with improvements on six of 20 statements, ranging from 8% to 40% increases. For confidence items, overall mean difference as well as individual item mean differences were significant for the pre-/retrospective pre- comparison. The overall mean and individual item mean for the pretest were higher than the retrospective pretest means. Course evaluations showed a score of 4.8 ± 1.2 of six regarding enhancement of knowledge on health literacy.	Assessed only for face validity; generalizability; lack of individual monitoring during role play which lacked a rubric; lack of use of standardized patients; confidence in their ability to create appropriate education materials for low literacy patients not tested

**Diaz-Cruz and Hagan (2020) [97]**	Assess the impact of a cultural continuum activity on the perceived cultural awareness knowledge of first-year pharmacy students	100 No demographics provided	Belmont University College of Pharmacy 90-min lecture within first-year student orientation program. Inclusive of a lecture, small and large group discussions, and debriefing	Investigator-design pre-post surveys including demographics, an item asking them define culture (pre-survey), items on perceived cultural awareness using a 5-point Likert scale (pre-survey), and items critically analyzing 12 statements as relevant to the cultural proficiency continuum, self-rating perceived knowledge using a 10-point scale (0 = not knowledgeable and 10 = very knowledgeable) (pre- and post-survey)	Pre-survey of perceived cultural awareness included: existence of stereotypes (4.72 ± 0.48), own personal biases (4.16 ± 0.75), how perspective influences judgment (4.09 ± 0.79), limitations of skills interacting (4.05 ± 0.87), feeling lack of capacity to respond appropriately (2.98 ± 1.30), provision of care being dependent on policies (4.21 ± 0.72), provision of care being dependent on approaches (4.11 ± 0.78). There were varying levels of recognition of statements related to cultural destructiveness (82.5–86.0%), incapacity (33.9–56.1%), blindness (36.8–82.5%), pre-competence (40.4–54.4%), competence (17.5–35.1%) and proficiency (75.4–80.7%). Self-rated perceived knowledge of the cultural proficiency continuum pre- and post-activity from 5.79 ± 2.48 (pre-) to 8.89 ± 0.92 (post-), p = 0.001.	Homogeneity of discussion groups due to self-selection; social desirability bias; lack of validation of instructor-developed statements; lack of post-survey assessment on cultural awareness

**Doutrich and Storey (2004) [37]**	(1) Identify strategies to improve students’ understanding and role performance in culturally competent population focused nursing and to identify barriers to improving performance, (2) Develop a stronger linkage and closer partnership between SWWHD and WSUV, (3) Enhance curriculum and faculty/SWWHD staff development to respond to changing trends in public health practice, and (4) Increase the number of WSUV baccalaureate students rotating through SWWHD and their total hours of clinical, and to introduce students to all SWWHD departments	13 100% RN to BSN students	Washington State University Vancouver College of Nursing and Southwest Washington Health District (now known as Clark County Health Department), “*Cultural Competence in Public Health Practice*” 16-week three-credit project to partner students with seasoned public health professionals to enhance the students’ cultural competence and improve their core public health knowledge and skills	Pre- and post-semester data from the 75-item CCTDI [138,139] and the 20-item IAPCC [100], pre- and post-semester. Narrative data were gathered from both students and mentors during an orientation for the clinical component of the course, during workshops/facilitated session, weekly post-clinical conferences, a reflective practice episode written paper, and a session reflecting on episodes from their years as mentors and preceptors.	IAPCC scores increased significantly (F = 8.37; df = 9; *p* = 0.018) from pre- to post-, and the CCTDI mean scores increased (314 to 320) non-significantly. On the post-, there were significant correlations (r = 0.685) between the CCTDI open-mindedness scale and the IAPCC scale, as well as significance (r = 0.663) between the CCTDI inquisitiveness scale and the IAPCC. Themes for strategies and barriers in the development of culturally competent population-focused nursing included: (1) open discussions of differences between acute and community practice, (2) articulating skills and knowledge of population-focused practice, (3) reflection and debriefing, (4) clarifying the differences between community and acute care, (5) the trusting connection, (6) decision making in public health, (7) getting the patient story, (8) identifying patterns and generalities in contrast to stereotyping, and (9) barriers to developing culturally competent, population-focused nursing.	Difficulty with mentors articulating practices; student lacked an understanding of the skills required; lack of control group; confounding factors

**Durand et al. (2012) [38]**	Increase students’ cultural competence by introducing them to different cultures and cultural characteristics that can affect patients’ health choices and outcomes	12 No demographics provided	Massachusetts College of Pharmacy & Health Science 10-week summer elective course in cultural competence offered to second professional year students	Pre- and post-semester 20-item IAPCC-SV [137] on culture.	Mean IAPCC-SV score improved from 57.1 to 68 (*p* = 0.022), with a significant increase in awareness (*p* = 0.027), desire (*p* = 0.01), knowledge (*p* = 0.0002) and encounters (*p* = 0.002) but not skill (*p* = 0.1).	Limited course length (10 weeks); difficultly implementing into the core curriculum; need for more resources, equipment, and faculty

**Fioravanti et al. (2018) [75]**	Combine cultural competency education, simulation, and educating students to use screening, brief intervention, and referral to treatment for alcohol and other drug use	183 85.5% women; 90.7% Caucasian, 6.6% Asian, 2.7% Hispanic, 1.6% African American, 1.1% American Indian; mean age 24.2 years (range 21–44)	University of Pittsburgh School of Nursing, *“ATN-SBIRT”* Embedded within psychiatric-mental health nursing course for junior-level nursing students. Used simulation to ensure all students encounter diverse patient situations followed by a debriefing session	Pre- and post- simulation 44-item CCA [103], as well as student opinions of the simulation experience and effectiveness as a tool for learning and for teaching cultural diversity.	Perception of culture awareness and sensitivity as well as cultural competence behavior increased pre- to post-simulation (p < 0.001). A total of 47% and 14% felt simulation was an effective or very effective learning tool pre-exercise, respectively, increasing to 72% finding it very effective post-expertise. 61% valued the simulation experience for teaching cultural diversity and 91% were more comfortable and able to apply culturally competent knowledge.	Lack of scripting in videos; lack of information of cultural differences at baseline; lack of assessment in clinical practice

**Fitzgerald et al. (2018) [83]**	Identify objective measures for growth in the development of intercultural competence for students who engage in education abroad programs of study using the IDI	10 90% women; age 18 to 21 years (10%), 22 to 30 years (90%); 20% lived in another country previously; 100% US citizens	The Ohio State University College of Nursing Three-credit elective education abroad course for sophomore or junior year traditional nursing students. Course included 7-week predeparture hybrid education and a 12-day service-learning trip to Nicaragua inclusive of work with a community partner health clinic in Ciudad Sandino	50-item IDI [111] (measuring an individual’s perceived and actual intercultural competence as defined on a continuum) in online format four weeks prior to departure and eight weeks after returning.	Participants’ Perceived Orientation scores were ‘Acceptance’ and Developmental Orientation scores indicated ‘Minimization’ for both pre- and post-departure. The Orientation Gap scores were significant, indicating that students’ perception overestimated their actual intercultural orientation; this gap narrowed over time (pre- 32.21 and post- 27.66). The Trailing Orientation score (pre- 3.22 and post- 3.60) indicated some participants were not fully resolved in terms of how connected or disconnected they felt. The Leading Orientation pre- and post-departure was ‘Acceptance through Adaptation.’	Sample size; students self-selected to participate; some missing data; generalizability

**Gibson et al. (2019) [93]**	Describe the process of incorporating patients as teachers of cultural sensitivity through a Cultural Sensitivity Panel, and to examine students’ self-awareness of their level of cultural competence after exposure to the panel	138 55% women; mean age 28 years	University of North Texas System College of Pharmacy, “*Cultural Sensitivity Panel”* Component of a required integrated pharmacotherapy course for third-year students, incorporating 5–8 presentations by panelists (of various cultural backgrounds and characteristics), focused by an interactive question/answer session	Investigator-designed pre-post surveys containing five items on knowledge perceptions and attitudes using a four-point Likert scale. Post-survey additionally contained demographics and two open-ended questions (regarding what was learned and who else should be included) to collect qualitative data.	For the five Likert items, scores improved between pre- and post- surveys for all (p < 0.05), including knowing the meaning of cultural competence (p < 0.001), feeling like education on this topic is important (p = 0.001), feeling like education on this topic will change behaviors (p < 0.001), feeling like the panel is worthwhile learning experience (p – 0.002), and feeling like the panel will change behaviors (p = 0.019). From the open-ended questions, key themes were learning about effective communication (64%), new resources for diverse patient populations (28%), addressing barriers to care (21%), the importance of patience and empathy (18%), and incorporating a patient’s background into their care (18%).	Single institution; lacking information on students’ other cultural experience or exposure; lack of tool validation; lack of information from the viewpoint of panelists; no assessment on outcomes as they translate to future experiential practice

**Giddens et al. (2012) [39]**	Assess the use of a virtual community as a teaching application to foster cultural awareness among nursing students	342 86.7% women; 55.4% white, 18.5% African American, 16.6% Asian, 9% Hispanic; mean age 24.4 years; 37.5% previous healthcare experience	Five schools (two on East Coast, one each in Midwest, Southeast and West; mixed public/private, with/without academic health sciences center), first semester fundamentals or skills courses within BSN programs, “*The Neighborhood*” – a virtual community with 40-character stories	Demographic survey and 22-item exit survey focused on four subscales (engagement in learning, cognitive outcomes, perception of usefulness, cultural awareness), frequency of use and open-ended questions. Subscale items (focused on cultural awareness) adapted from CSI, a component of the Flashlight Evaluation System (from [140]).	Mean cultural awareness subscale score was 3.58 ± 0.69, with range 1.33–5.0. Scores on cultural awareness subscale differed depending on use of the community: no use (3.39 ± 0.69), low use (3.52 ± 0.7) and high use (3.81 ± 0.57). Correlation was found between level of use and cultural awareness (r = 0.246; *p* < 0.0001).	One subscale of data available; lack of consistent use of community across faculty

**Gonzalez et al. (2015) [40]**	Enhance students’ knowledge, attitudes, and self-confidence in addressing health disparities	39 62% women; 38% non-Hispanic white, 28% Asian, 15% Hispanic, 10% non-Hispanic black; median age 25 years; 74% natural science majors, 10% social science, 15% humanities	Albert Einstein College of Medicine 13-session elective for first-year students using the guidelines for health disparities education curricula created by the Society of General Internal Medicine’s Disparities Task Force	Investigator-designed pre- and post-test with five open-ended knowledge items, four attitude items, three confidence items and one skills item using four-point Likert-type scale	Total knowledge increased from 15.9 ± 2.5 to 19.1 ± 3.2 (*p* < 0.01) from pre- to post-survey. Self-reported confidence (as measured by knowledge and skills) also increased from 10.7 ± 1.5 to 14.4 ± 1.7 (*p* < 0.01). Attitudes improved from 16.7 ± 1.9 to 18.2 ± 1.1 ( *p* < 0.01). Younger students ( < 24 years) had a greater change in confidence.	Self-reporting and lack of observation; no comparison group; correlation of individual sessions to outcomes

**Gonzalez et al. (2014) [41]**	Describe an educational intervention addressing both health disparities and physician implicit bias and the results of a subsequent survey exploring medical students’ attitudes and beliefs toward subconscious bias and health disparities	218 56% white, 5% Latino, 6% African American, 22% Asian,	Albert Einstein College of Medicine, “*Disparities in Health and Health Care: Impact on Patients and the Role of Doctors*” Session part of longitudinal third-year course spanning the various clinical clerkships	IAT [117] and investigator-designed 15-item survey focusing on results from the IAT using a four-point Likert scale.	A total of 65% of students reported a preference for people like themselves, with this preference more common among the deniers than acceptors (87% vs. 60%, *p* = 0.00027). More deniers disagreed with “Health disparities exist in the United States,” (9% vs. 1%, *p* = 0.02) and were more likely to agree that “Doctors treat all patients the same, no matter what ‘group’ they belong to,” (19% vs. 7%, *p* = 0.01) and “The US health care system is fair and equitable and provides ‘blinded’ care” (23% vs. 9%, *p* = 0.0075). Deniers were less likely to report observing nurses treating patients differently based on race, ethnicity, or other similar factors (41% vs. 62%, *p* = 0.01).	Confounding of previous exposure to topics of health disparities and implicit bias; lack of validation, small sample; differences across group sessions; non-response bias

**Hawala-Druy and Hill (2012) [42]**	Design and implement eclectic, creative, evidence-based interdisciplinary educational activities, along with culturally congruent teaching strategies, within a semester-long university course that promoted positive and culturally competent learning outcomes for culturally diverse, largely millennial students	106 80.6% women; 54.1% < 23 years of age; 42.9% pharmacy, 32.7% nursing, 24.5% allied health; 82.5% African American, Black, or Caribbean	Howard University, “*Culturally Congruent Care for Clinical Health Professions*” Three-credit interdisciplinary elective	IAPCC-SV [137] pre- and post-semester on culture. Course evaluations, student feedback and portfolio reflections were also collected.	Significant changes in IAPCC-SV scores pre- and post- (60.8 to 70.6; *p* < 0.001). Significant changes were also seen across stratifications for gender, profession, ethnicity, and class cohort. Analysis of portfolio/journal reflections found an increase in sensitivity and competency, and satisfaction with the instructional methods.	Unable to match all pre- and post-surveys; small subgroup sample sizes; lack of formal qualitative analytic method

**Heffernan et al. (2013) [43]**	Determine the effectiveness of cross-cultural field excursion initiatives as a means to encourage cultural competency in student pharmacists	17 53% women, 30% Caucasian, 30% Hawaiian/part Hawaiian, 5% Hmong; 82% aged 22–30; 35% first professional year, 35% second professional year, 30% third professional year; entire/majority of life living in Hawaii 35%	University of Hawai’i at Hilo College of Pharmacy Excursion to Kalaupapa, Molokai, to visit an isolated Hansen disease settlement	Pre-trip research paper and post-trip reflection presentations. Focus groups were conducted where students shared the most interesting experiences, lessons learned, importance of cultural competency to provide pharmaceutical care, insights in social and cultural foundations of Hawaii, local perspectives on healthcare, usefulness of field excursion to increase cultural awareness and sensitivity, changes in pharmacy practice philosophy, value added to didactic course work and how knowledge will assist future	Major themes included: (1) impact of experiential learning (classroom vs field excursion), (2) cultural knowledge (Hawaiian values/beliefs and world views, shifts in cultural perspectives, culture of disease, politics and public health policy and effects of stigma), (3) attitudes, reflections, and shifting perspectives (compassion, empathy, humility, self-reflection, sensitivity to others) and (4) application to pharmacy profession (utilization of knowledge gained).	The generalizability of Hansen disease, difference in theorizing about clinic practice and actual practice skills, the lasting impact is unknown and the fact that the development of cultural competence is devoid of a distinct end point.

**Hunter and Krantz (2008) [44]**	Reports the findings of pre- and post-assessments of the multiple constructs of cultural competence, as delineated within the Process of Cultural Competence in the Delivery of Healthcare Services model	76 68.4% in summer course, 31.6% fall course; 100% in master’s level tracks for adult, family, pediatric, neonatal, or women’s health nurse practitioners; majority Caucasian	University of Missouri-Kansas City Graduate-level course with units based on four of five constructs by Campinha-Bacote, [141] including cultural awareness, knowledge, skill, and encounters.	25-item pre- and post-assessment using IAPCC-R [137] on culture.	Scores improved on all constructs from pre- to post-assessment, including awareness (74.5 to 86.7), knowledge (55.1 to 71.4), skill (61.2 to 83.8), encounters (65.2 to 79.5), desire (83.2 to 92.0), and the overall competence score (68.2 to 82.8, all *p* < 0.001). No differences in live vs. online offerings of the course.	Most significant change was in cultural desire, the construct that was not directly “taught” in the course

**Isaac et al. (2015) [45]**	Examine linguistic differences in dental students’ reflective writing assignments before and after interviewing an individual who was culturally different from themselves	80 55% women; 32% underrepresented minorities, 68% white, non-minority	University of Florida Course in behavioral sciences for first year students	Linguistic inquiry and word count (calculates the degree to which people use various categories of words) for two reflective essays, once which described their personal awareness for race, gender, ethnicity, social class, sexual orientation, mentally or physically challenged, faith, and cultural groups, and one which designated what type of individual they were to interview.	There was a significant difference between essays which indicated increases in words and phrases (1) concerning mindfulness of other’s influence, (2) concerning inclusivity and relatedness and (3) illustrating a willingness to admit being wrong in assumptions.	Influence of lectures on cultural disparities, ethics, and social justice between the essays; priming from writing or social acceptability to the instructor.

**Jarris et al. (2012) [46]**	Evaluate the efficacy of a newly reformed curriculum for teaching culturally responsive care and to build awareness of health and health care disparities in first-year medical students	136 52% women; 71% white, 22% Asian, 3% African American, 2% Native Hawaiian or Pacific Islander, 2% American Indian or Alaska Native, and 3% Hispanic/Latino	Georgetown University School of Medicine, “*Social and Cultural Issues in Health Care*” Required course for first year students	Validated pre- and post-tests adapted from the *Provider’s Guide to Quality and Culture* (from [142]) and the National Center for Cultural Competency’s *Self-assessment Checklist for Personnel Providing Primary Health Care Service* (from [143]). Items on awareness of inherent biases and cultural responsiveness (five items) using Likert scale questions, as well as cultural knowledge (seven items).	For the awareness-based questions, scores improved on three of five questions, including “*I keep abreast of the major health concerns and issues for ethnically and racially diverse populations residing in the geographical locale in which I am currently residing*,” “*I am aware of the socioeconomic and environmental risk factors that contribute to the major health problems of culturally, ethnically, and racially diverse populations in the Washington, DC area*” and “*Even though my professional or moral viewpoints may differ, I accept that individuals and families are the ultimate decision makers for services affecting their lives*.” (all *p* < 0.05). Scores increased for six of seven knowledge-based questions, with the largest improvement for “*Lower rates of kidney transplantation for African American patients compared to whites are explained primarily by differences in*…”	Variability of student exposure to cultural competency training; preconceived attitudes; inadequate clinical exposure for context

**Kamau-Small et al. (2014) [47]**	Report on the evaluation process of a multi-disciplinary interactive teaching-learning workshop implemented in a college of nursing baccalaureate program	149 No demographics provided	University of Colorado at Colorado Springs Senior level community/public health undergraduate nursing course (25–40 students per semester)	12-item quiz (on knowledge), two online surveys (at two- and eight-weeks post, on perceptions), and a clinical application report. Survey questions were adapted from the Transfer Barriers Framework (from [122]) and the Stages of Change (from [144]) was applied as an audit tool for qualitative data.	100% of students scored eleven or twelve points on the (open-book) knowledge quiz. Two-week survey identified the theater pieces (23%) and interactive activities (22%) within the workshop as most valuable; 83% of students indicated an increase in awareness, 13% reported evidence of behavior change and 4% indicated a change in behavior. At the eight-week survey (post-clinical experience) 58% reported change in awareness and 28% reported change in behavior. Among clinical application reports, 25% reported precontemplation, 37% indicated contemplation and 31% indicated determination to change.	Lack of formal research design and pre-workshop baseline assessments; self-reported data; only used a subset of weekly clinical application reports

**Knecht et al. (2018) [73]**	Explore whether participation in diverse clinical settings in an ethnically diverse inner city that serve the underserved followed by guided critical reflection would significantly increase students’ level cultural competence as measured by the IAPCC-SV	58 (25 intervention, 33 control) 100% women; mean age 23 years (range 21–38); 4% (intervention) and 3% (control) Black, 8% (intervention), and 6.1% (control) Hispanic, 4% Asian (intervention), 4% Indian (intervention)	University of Saint Joseph Part of a senior-level community health course for undergraduate nursing students, with students participating in traditional clinical placements (control) or in diverse clinical settings with the underserved (intervention)	20-item IAPCC-SV [137] upon curriculum completion as well as participation in a focus group.	Mean IAPCC-SV scores were 68.71 ± 4.9 (intervention) vs. 58.86 ± 3.2 (control), p < 0.001, with significant differences for all constructs except awareness. Significant increase (intervention vs. control) was seen for knowledge (15.9 ± 2.1 vs 12.7 ± 1.6; p < 0.001); skill (9.3 ± 1.4 vs 7.3 ± 1.0; p < 0.001); encounters (17.4 ± 1.9 vs 15.0 ± 1.2; p < 0.001); and desire (15.2 ± 0.7 vs 13.2 ± 1.1; p < 0.001). Themes identified from focus groups included enlightenment, competence, and connection.	Single school; selection bias; influence of independent student demographic attributes or cultural experiences

**Knockel et al. (2019) [92]**	Design and implement an active learning activity for second-year students, and assess its effectiveness in increasing student knowledge and comfort level in caring for a transgender population	85 No demographics provided	University of Iowa College of Pharmacy Activity on caring for patients in the LGBTQ community within a required practice skills laboratory for second-year students, alongside co-enrollment in a genitourinary/reproductive therapeutics course	Investigator-designed pre-post assessment using three items to assess knowledge on use of hormone therapy for transgender patients and four questions to assess comfort in caring for transgender patients (using a five-point Likert scale). Post-survey included two additional activity evaluation questions as well as an open-text field to share opinions.	For knowledge-based questions regarding transgender health, scores improved (p < 0.0001) from pre- to post-survey, including on testosterone route of administration (82.5 to 97.5%), use of estrogen in the setting of deep vein thrombosis (22.5 to 90.0%) and preferred estrogen products in transgender women (10.0 to 65.0%). On the post-survey, 96.5% strongly agreed/agreed they understood the role of the pharmacist, 70.6% felt comfortable caring for transgender patients, 83.5% felt comfortable with appropriate technology when counseling, 76.4% feel the program has adequately exposed them to information to fill these roles. The open-text responses resulted in two primary themes related to activity strengths and opportunities for improvement.	Quiz not administered in real-time; limited depth of knowledge assessment; lack of measurement of long-term retention of information; lack of information on whether didactic training improves practice

**Leach et al. (2019) [96]**	Measure the general perceptions and attitudes of pharmacy students toward transgender patients and health and evaluate students’ level of support for receiving education in transgender healthcare	60 No demographics provided	University of Maryland, *“Gender Transition Therapeutics”* One-hour lecture part of a required second-year Endocrinology, Women’s Health, and Genitourinary course for pharmacy students. Lecture included Content included therapeutics, cultural competency, critical consciousness, and language related to care for transgender individuals	Investigator-designed 5-item pre-post survey to evaluate perceptions of managing transgender patients (using a 5-point Likert scale), with three additional multiple-choice questions assessing knowledge post-lecture.	Overall mean perception score increased from 2.8 to 3.91 pre- to post-lecture (p < 0.0001). Confidence in managing health concerns (2.1 to 3.1; p < 0.01), awareness to gender neutral pronouns (2.8 to 4.1; p < 0.01), awareness of barriers faced (3.3 to 4.2; p < 0.01), and awareness of guidelines (1.9 to 4.0; p < 0.01) increased. Students strongly agreed at baseline that pharmacists play an important role in promoting transgender health.	Response bias, non-validated tool, limited intervention without opportunity to practice/reinforce

**Leeet al. (2016) [48]**	Describe curricular components of a service learning project that may potentially enhance post-baccalaureate student perspectives of humanism in medicine	41 53.7% women; 80.5% Hawaiian origin, 17.1% Guam, 2.4% American Samoa; 65.9% rural origin, 29.3% ESL; 22.0% Hawaiian ethnicity, 29.3% Filipino, 31.7% Asian, 12.2% Chamorro; 63.4% indigenous; mean age 26 years; 100% pre-medical students;	University of Hawai’i at Mānoa School of Medicine, “*Imi Ho’la’s Scientific Basis of Medicine*” Post-baccalaureate course where the Kalaupapa service learning project is the capstone, with a three-day immersive experience in Kalaupapa, Moloka’i.	Narrative comments extracted from student reflection essays in response to the service-learning project that covered (1) discuss the role of disease and impact on individuals, family, community, and society, (2) what was learned about yourself, (3) what learned about others, and (4) improvements for future trips.	Themes identified included: (1) sense of loss and resulting isolation, (2) unity, (3) resilience, (4) awareness of self and others, (5) reinforced future professional goals, (6) teamwork, (7) fellowship, (8) future, (9) length of stay, (10) level of interaction, and (11) gratitude.	Small sample size; limited prompts; potential bias for thematic reviewers; no baseline data regarding the students’ knowledge and attitudes of humanism

**Leung et al. (2016) [49]**	Share the opportunities, mechanics, and challenges characterizing the experiences of student leaders in pre-clinical course development	18 No demographics provided	Brown University Warren Alpert Medical School, “*Healthcare for the Underserved*” Pre-clinical elective with five modules on: (1) refugee/immigrant health and medical-legal issues, (2) child obesity and the built environment, (3) hypertension rates and race/ethnicity, (4) teen pregnancy and sex education, and (5) mental health and homelessness	Investigator-designed quantitative/qualitative feedback assessment on student experiences via five-point Likert scale after each class and course. Supplemented by a nine-item one-time pre- and post-course survey with four-point Likert scale on knowledge and attitudes.	Overall pre-post survey score increased from 3.26 to 3.41 (*p* < 0.02). A significant improvement was seen for the individual item “*I have a good understanding of what “underserved” means*,” but the other statements had non-significant changes.	Translation of attitudes to real life; limited changes in attitudes due to selection bias; limited data

**Levey (2020) [91]**	Assess changes in graduate nursing students’ perceptions of cultural competence and cultural nursing practices as an outcome of completing an online multi-perspective course on cultural diversity	37 78.4% white, 5.7% Black/African American, 2.7% Asian, 2.7% Hispanic/Latino, 5.4% Other; mean age 35 years; mean years as a registered nurse 8.5 years; nurse practitioner program 81%, nurse educator program 13.5%	Carthage College, “*Culturally Congruent Care for Advanced Nursing”* 16-week online course focused on the cultural domains of ethnic (Hispanic, Native American, white, Black, and Pacific Islander/Asian American cultures) and nonethnic (vulnerable perspectives based on life context or choices [e.g., veteran, military, sex worker, disability, homeless, parents who refuse to immunize, HIV/AIDS, cancer survivors, LGBQTIA, or victims of intimate partner violence]) populations in the US	Investigator-designed CCCAN-CLO instrument, including seven items on attainment of cultural practices using a 5-point Likert scale. Also utilized the validated CCA [145], inclusive of two-subscales on cultural awareness and sensitivity (11 items) and cultural competence behaviors (14 items).	Statistically significant increase on cultural practice (CCCAN-CLO mean 22.67 [4. 76] to 27.75 [3.^68^], p < 0.001), cultural awareness and sensitivity (mean 47.45 [3.^95^] to 50.10 [3.^78^], p < 0.001) and cultural competency behaviors (mean 52.51 [8.^29^] to 59.16 [6.^84^], p < 0.001). The largest change was for CCCAN-CLO scores related to cultural practices in healthcare settings, followed by cultural behaviors, awareness, and sensitivity.	Generalizability; use of five-point Likert-type scale causing a ceiling/floor effect in survey responses; CCCAN-CLO factor not confirmed with advanced psychometric testing; no objective measurement of knowledge of cultural awareness, sensitivity, behaviors, and practices; no measurement in actual clinical practice

**Limet al. (2008) [50]**	Measure the effectiveness of a presentation designed to increase cultural competence by teaching cultural knowledge about specific ethnic groups; to encourage positive, nonstereotypical attitudes toward patients with limited English proficiency; and to introduce first-year students to interpreting services and teach them interpreting skills	63 50.8% women; 44% Caucasian, 41.3% Asian American; 44% monolingual; mean age 24.5 years; 74% born in the US; 50.8% no interpreting experience	University of California Davis School of Medicine, “*Cultural Issues in Medicine-Cultural Competence, Patient Expectations, and Using Interpreters*” Two-hour presentation for a first-year medical student in a course on behavioral sciences and the doctor-patient relationship.	Investigator designed nine-item questionnaire on a seven-point Likert scale with four items on attitudes and five on knowledge	Pre- and post-intervention scores on composite attitude and knowledge domains were significantly different (no other specific data provided).	Lack of numerical results, limited number of items; lack of comparison group; attitude items misconstrued as beliefs or opinions

**Liu et al. (2015) [51]**	Describes an evaluation of the perceived impact of the IPE sessions using the revised Clinical Cultural Competency Questionnaire (CCCQ) and a knowledge quiz	160 Men:women 1:6 (nursing) and 1:2 (pharmacy ); 50% nursing, 50% pharmacy	Southern Illinois University Edwardsville School of Nursing, School of Pharmacy and Department of Applied Communication Studies in the College of Arts and Sciences Second-semester required course that focused on health assessment (nursing) and a required course on cultural competency and health literacy (pharmacy)	Revised/shortened 18-item CCCQ (from [146]) to assess changes in self-perceived knowledge, skills, attitudes and encounter comfort level using five-point Likert scale. Eight items measured the participants’ perceived skills, three items measuring perceived knowledge, six items measuring comfort levels with encounters/situations and two items measuring the participants’ attitude.	Actual knowledge (6.724 to 8.723) and all four CCCQ subscales (skills, perceived knowledge, encounter comfort and attitude) significantly increased pre- to post-intervention. Statistically significant differences were detected in actual knowledge, F(1,137) = 32.880, *p* < 0.001; and on the attitude subscale, F(1,137) = 6.803, *p* < 0.01, between nursing and pharmacy students (pharmacy students scored higher in actual knowledge, nursing students scored significantly higher in attitude).	Only two sessions conducted; intercultural competence training limited in scope; single course intervention; lack of control group

**Lonneman (2015) [52]**	Describe the use of six teaching strategies as an intervention designed to promote growth in nursing students’ cultural awareness	34 experimental and unknown control Age range 20–50 years; 100% bachelor’s degrees	Mount St Joseph University Population-focused community health course during second semester of four-semester MSN program	Pre- and post-assessment using the third section (30-items) of the TSET [101], using a 10-point Likert scale. Investigator-designed survey to rate experiences using 10-point Likert scale and comments. Intervention group tested again eight months post-program.	Students in the intervention group gained 25.8 ± 29.9 points from pre- to post- (8.6% increase), while the change in the control group was 17.1 ± 36.6 (5.7% increase, *p* = 0.054). Scores for intervention group at eight months increased seven points (2.8%). Students rated experiences as overall effective (6.8 to 8.2 of 10).	Scope; diversity of students; lack of connection between perceived improvements and clinical effectiveness

**Mainet al. (2013) [53]**	Explore the lived experience of BSN and MSN students participating in a multidisciplinary service-learning course in a rural, underserved village in Belize	9 No demographics provided	Western Kentucky University International Multidisciplinary Public and Clinical Health Team (IMPACT) project	Student journals (open-ended with suggested topics) before, during and after trip	Eight themes emerged: (1) expectations and emotions regarding trip; (2) developing a reciprocal relationship with the community; (3) valuing interdisciplinary collaboration; (4) acquiring knowledge that would impact their future nursing practice; (5) growing personally; (6) making future plans to continue service work; (7) recognizing themselves as part of a larger social network and a shared responsibility for social problems; and (8) buying into interdisciplinary change projects	Small sample size, lack of theme correlation with time frame of journal entry (before, during or after)

**Matsunaga et al. (2003) [54]**	Report on a qualitative evaluation of a community-based model for teaching cross cultural competence	120 No demographics provided	Kalihi-Palama Health Center (a community health center in downtown Honolulu serving an underserved multiethnic population), “*Kalihi-Palama Community Health Seminar*” Two-semester community service-learning course developed in collaboration with a neighborhood elementary school	Weekly journal entries (not factored into evaluation/grades), submitted four times during the year for review by the faculty.	Cultural themes included: (1) gaining insight: examining one’s own culture and values; (2) understanding the interaction of culture and health; (3) learning to ask: eliciting client and community perspectives; (4) working with differences in a diverse healthcare team; and (5) learning by teaching: practical engagement with community residents	Data analyzed by course faculty; social desirability bias on the part of students

**McCave et al. (2019) [98]**	Describe a team-based interprofessional education simulation activity developed as a teaching activity at a university for graduate health care learners	494 33% OT, 22% PA, 17% medicine, 13% PT, 8% social work, 7% nursing	Quinnipiac University Team-based interprofessional education simulation activity including panel discussion, discharge planning with SPs, team huddle, team debrief, video simulation, debriefing as a large group	Investigator-designed post-activity electronic survey assessing the value of event components, items on four IPEC competencies (values and ethics; role and responsibilities; interprofessional communication; teamwork) adapted from [147].	≥90% strongly agree or agree on all the activities and outcomes related to the IPEC competencies in 2017 and ≥80% in 2018. Th highest was for “respect the unique cultures…” with 93% overall and the lowest was “give timely and sensitive, and instructive feedback to others…” at 86%.	Resource-intensive to develop workshop; lack of all health professions; limited number of years of data; single school

**McElfish et al. (2017) [77]**	Improve participants’ knowledge, attitudes, and behaviors related to working with other cultures (specifically, the Marshallese) and other health professions.	98 57.7% women; mean age 29.4 years; 26.9% medicine, 30.8% nursing, 30.8% pharmacy; 91.5% European/Caucasian; 100% English as primary language	University of Arkansas for Medical Sciences Northwest Interprofessional education program with educational seminars, service-learning, cultural exposure, and competence-building activities for student teams	Modified versions of the 28-item CCCHS [105] and the 19-item RIPLS [121] were used in pre-post design alongside demographic items and open-ended items about the program, in addition to focus groups.	Post-program RIPLS scores Teamwork & Collaboration subscale (39.63 ± 5.42) and Professional Identity subscale (28.10 ± 4.43) increased from pre-program scores. Post-program CCCHS scores (59.87 ± 12.63) increased significantly from pre-program (48.57 ± 17.31, p < 0.001). Within focus groups, emergent themes included change in knowledge, change in attitudes, and change in behavior.	Reliance upon self-reported perceptions; focus groups held per profession; low participation in focus groups; lack of participant demographic data; single school in specialized area

**McEvoy et al. (2009) [55]**	Describe a session, ‘‘Cross Cultural Communication-Using an Interpreter’’, honed over the past three years to equip students with knowledge and skills to use interpreters	110 No demographics provided	Albert Einstein College of Medicine, “*Patients Doctors and Communities*” 16-session longitudinal course during third-year clerkships	Investigator-designed three-item retrospective pre-post survey on perceived self-efficacy. Course evaluations were also conducted.	77.3% agree (communicate with patients with limited English), 77.3% agree (give instructions to untrained interpreter) and 76.4% agree (access a hospital language line). Course evaluation scores ranged 4.1 to 4.4 of five for accomplishment of session objectives across three years of offering.	Low quality assessment tool; outside influence of clinical experiences

**Miller and Green (2007) [56]**	Examine medical students’ reflections on a cultural competence OSCE station as an educational experience	22 64% women; 45% Caucasian, 23% Black/Hispanic, 32% Asian-American; 27% reported ‘doing well’ on station, 59% reported ‘feeling challenged’ by station, 14% doing “poorly” or “failing” station	Harvard Medical School, “*Patient Doctor II*” Required course for second-year students	One-on-one semi-structured interviews	Take home points included: (1) the importance of eliciting the patient’s perspective on their illness in a culturally sensitive way; (2) the need to examine how and why patients take their medications and to inquire about complementary and alternative therapies; and (3) the importance of exploring the range of social and cultural factors associated with medication nonadherence, Knowledge and skills acquired: (1) exploring medication non-adherence in greater depth; (2) asking about the patient’s perspectives and social context; and (3) inquiring about sensitive issues such as literacy and immigration status.	Small, self-selected sample; inability to stratify results among students

**Mkandawire-Valhmu et al. (2019) [84]**	Develop an understanding of how watching documentaries in a cultural diversity course helped nursing students gain perspective on nursing care of diverse populations	90 No demographics provided	University of Wisconsin-Milwaukee Three-credit semester-long course across two campuses for first- and second-year nursing students on cultural diversity and cultural safety	Thematic evaluation of student reflections to two the viewed documentaries (“God Grew Tired of Us” and “Rape in the Fields”) using Krathwohl’s Taxonomy of the Affective Domain for framework	Themes identified reflected the taxonomy levels in the framework. Students routinely reflected at the lower levels of the taxonomy, for example: receiving (openness to learning); responding (students showed empathy for others but could not connect those experiences to societal factors across settings); valuing (students re-evaluated their stereotypes). Few students reached higher levels of the taxonomy	Selection bias; themes not clearly defined separately from the stages of taxonomy; single school; single year; data only from 2/9 documentaries discussed in class

**Ndiwane et al. (2017) [71]**	Implement an OSCE educational innovation to improve cultural humility and assess pre- and postintervention effects on students’ knowledge and awareness of cultural diversity	63 90.5% women; mean age 21 years (range 20–38 years); 64% no previous experience/course in cultural humility	MGH Institute of Health Professions Activity within an adult gerontological advanced health assessment class for Master’s in Science in nursing students. Students reviewed an exemplar video, conducted/reviewed a video of their health history encounter with a SP	Pre- and post-assessment with 17-item CAS [106] on awareness of cultural diversity and biases regarding issues/practices. Also included survey for demographics and open-ended evaluation questions, and the 10-item Student Satisfaction Survey, [148] with subscales in satisfaction, self-confidence, and critical thinking.	Knowledge scores increased from pre- to post-assessment (2.44 ± 0.75 vs 2.97 ± 0.60; p = 0.001). Scores for satisfaction (3.83 ± 0.67), self-confidence (3.19 ± 0.78), and critical thinking (3.59 ± 0.76) were statistically significant (p < 0.001).	Limited qualitative analysis; single school; small sample

**Nokes et al. (2003) [57]**	Develop and refine a 15-hour service-learning intervention and explore whether participation in the intervention made a difference in the critical thinking, cultural competence, and civic engagement of nursing student participants	14 100% women; 56% Caucasian, 31% African American, 13% Asian; mean age 39.6 ± 9.0 years; 69% undergraduate nursing program; 56% no experience in community nursing	City University of New York 15-hour intervention with 6-hour classroom intro seminar, seven hours online interactive program, and a two-hour classroom summary seminar.	75-item CCTDI, [149] consisting of seven subscales: inquisitiveness, systematicity, analyticity, truth-seeking, open mindedness, critical thinking, self-confidence, and maturity. 20-item IAPCC [137] on culture. 12 items on civic engagement were adapted from [150].	Critical thinking scores decreased post- (t = –2.23, *p* = 0.04), particularly on the self-confidence subscale (t = 2.29, *p* = 0.039). Except for post- self-confidence scores, all of scores were higher than 40, which indicates average to consistent endorsement of that disposition of critical thinking. Cultural competence scores were significantly lower (53.16 ± 5.28 to 41.57 ± 5.65, *p* < 0.001) post-, but civic engagement increased (40.42 ± 4.29 to 44.30 ± 4.79, *p* = 0.004). Further analysis on the 6 items for found a significant increase on this subscale post-(t = –3.097, *p* = 0.009).	No control group; small sample size and limited recruitment

**Olukotun et al. (2018) [80]**	Gain insight into how pre-clinical nursing students’ worldviews about difference are formed, changed, and expanded in the context of an undergraduate cultural diversity college course; understand what value students gained from the course and how the content was helpful in changing their perceptions	90 No demographics provided	University of Wisconsin-Milwaukee Three-credit semester-long course across two campuses for first- and second-year nursing students on cultural diversity and cultural safety	Thematic evaluation of mid-term and final reflection papers using Krathwohl’s Taxonomy of the Affective Domain for framework.	Themes identified reflected the taxonomy levels in the framework, for example: receiving (acknowledging their held stereotypes); responding (moving beyond awareness to expressing their reaction towards injustice); valuing (appraisal of the values taught in the course to internalization of these same values); organization (students described grappling with values of their families vs new values learned), and characterization (students provided examples of ways that new values around diversity have been incorporated into their lives.	Selection bias; themes not clearly defined separately from the stages of taxonomy; single school; single year

**Omoruyi et al. (2017) [78]**	Determine if structured curriculum on interpretation would promote learners self-reported competency in these encounters and if proficiency would be demonstrated in actual patient encounters	206 (176 intervention and 30 control) No demographics provided	University of Texas Health Science Center at Houston McGovern Medical School Educational intervention on interpreters and use of communication technology during third-year clinical rotations	Pre- and post-training (8 weeks) competency self-assessment using 10-point Likert scale (novice to proficient), as well as a modified FORS [110] assessment of audio files of telephone interpreter interactions.	Improvements were seen in competence/awareness from pre- to post-assessment for assessing need for an interpreter (7.21 to 8.68), maintaining accuracy with telephone interpreters (6.55 to 8.10), how to use telephone interpreters at high quality (6.35 to 8.19) and caring for limited English proficiency patients (6.57 to 8.36, all p < 0.001). For FORS assessment of audio, the intervention groups had higher baseline and maintained scores for their rotation, whereas the controls had fluctuation and inconsistency in scores.	Single school; single class year; generalizability; lack of capacity to understand longevity of skills

**Ottet al. (2004) [58]**	Explore the development of nursing students’ cultural competence within the context of an urban service-teaching project that required reflection on their experience with racially diverse urban high school students	28 82% women; 100% white; mean age 23.7 years; 96.4% attended public high schools with limited racial diversity; 17.9% attended urban high schools	University of Wisconsin-Milwaukee, students Two-credit health promotion course to conduct a teaching project on substance abuse prevention with high school students	Investigator-designed debriefing session between presentations, written exit survey and theoretical paper at end of the project and focus group interviews four-months post-project.	Transformations experienced included: (1) questioning stereotypes; (2) reflecting on differences; (3) gaining the know-how to interact; (4) understanding the power of one; and (5) imagining and taking action.	Lack of connection between project factors and changes seen

**Ozkara San (2019) [85]**	Examine the effect of the DSPS cultural competence education strategy on students’ transcultural self-efficacy	53 41.8% women, age < 25 years 37.7%, 25–30 years 11.2%, 30–34 years 3.6%, 35–39 years 1.2%, 40–44 years 0.0%, 45–49 years 1.2%; single 46.9%, living with partner 3.6%, married 4.7%; white 32.6%, Asian 7.13%, Black/African American 4.8%, Hispanic/Latino 6.11%, multiracial 2.4%, other 2.4%; English as first language 41.8%; Fluent in language other than English 20.4%	Pace University Lienhard School of Nursing Two simulation scenarios within a second-semester nine-credit 15-week medical-surgical nursing course videos, assignments, interactions with culturally diverse SPs and assignments	Pre- and post-test design using the 83-item TSET, [101] as well as demographic data and additional investigator-designed correlation measures.	TSET self-efficacy strength score changes from pre- to post-test improved for cognitive (7.3 to 8.33) and practical (7.61 to 8.33) subscales with only the affective subscale not statistically significant (8.83 to 9.14; p = 0.054). Total score improved from 7.96 (pre-) to 8.62 (post-), p < 0.05. Distribution of changes between the pre- and post-test self-efficacy levels on the cognitive (p = 0.003) and practical (p = 0.019) subscales were significant, but not on the affective subscale (p = 0.058).	Convenience sample; small sample; generalizability; lack of control group; difficult to control for confounders; use of non-validated measures

**Parkhill et al. (2014) [59]**	Examine the impact of a panel discussion on transgender health care on first-year pharmacy students’ knowledge and understanding of transgender experiences in an Introduction to Diversity course	78 No demographics provided	St John Fischer College, “*Introduction to Diversity*” Required (pass/fail) course during first professional year	Survey instrument developed by partner (Gay Alliance of Genesee Valley) on content area, usefulness of information, knowledge of presenters and overall evaluation (on a five-point Likert scale), investigator-designed survey instrument, student reflections on panel discussion	GAGV survey showed high evaluations (ranging 4.5 to 4.7 of five) for the four areas. Self-perceived knowledge about LGBT issues increased significantly after the presentation (students who had reported “none” or “a little” knowledge decreased to 0% and reports of knowing “a lot” increased to 86%). Themes regarding the single most important item learned included: (1) optimizing interactions with transgender patients in the pharmacy; and (2) understanding the transgender population. Investigator survey showed moderate to high achievement of learning outcomes (3.1 to 4.4 of five). Major themes in reflections included: (1) eye-opening to challenges; (2) empathy and understanding; (3) knowledge and desire to learn more; (4) appropriate healthcare; and (5) communication.	Overambition of learning objectives achieved; preconceived notions

**Peluso et al. (2018) [76]**	Describe the impact of an open-access, case-based global health ethics workshop and the breadth of dilemmas faced by students to inform future interventions	82 93% previously traveled outside of the USA; 35% born/lived outside the USA; 22% had previous ethics training	Yale University School of Medicine 90-min case-study, discussion-based session about ethical scenarios in global health for students participating in funded global health clinical electives	Investigator-designed pre-post electronic surveys incorporating closed-ended five-point Likert scale items as well as open-ended items	From pre- to post-activity, there were perceived improvements in preparedness to manage ethical dilemmas while abroad (3.6 vs. 4.4; p < 0.001), to describe domains of ethical dilemmas (3.2 vs. 4.5; p < 0.001), to identify ways to prepare for ethical dilemmas (3.0 vs. 4.2; p < 0.001), to identify contacts if they faced dilemmas (2.7 vs. 4.3; p < 0.001), and to identify strategies for managing themselves during dilemmas (3.3 vs. 4.4; p < 0.001). Mean perceived likelihood of facing an ethical dilemma while abroad increased from 85% to 95% (p < 0.001) pre- to post-activity. Students provided free responses (100 before workshop, 85 after workshop and 60 of returning students) of discrete examples of ethical dilemmas.	Single school; missing data; small sample size; influence of unknown biases; non-response bias; persistence of perceptions over time; no incorporation of moral relativism

**Perryet al. (2003) [60]**	Assess the impact of the elective course on student’s level of confidence in organizing future public health efforts and providing population-specific pharmaceutical care	Eight No demographics provided	University of Louisiana at Monroe College of Pharmacy, Medical Outreach Experience 13-week (10-week training and preparation, one-week international experience, post-outreach reflection), two-credit hour elective course for second- and third-year students	Investigator-designed 20-item survey regarding confidence in organizing medical outreach (seven items) and confidence in providing pharmaceutical care using a five-point Likert scale. Post-course evaluation survey with both closed- and open-ended items was also conducted, including nine items on course value, and 13 items on different educational components.	Five of seven items for organizational components significantly improved from pre- to post- (*p* < 0.05), while three of 13 pharmaceutical care components improved (*p* < 0.05). Responses to the course evaluation indicated that students found high value in the course. Themes from the open-ended questions included: (1) opportunity to gain a different perspective of the practice of pharmacy, specifically as it related to the value of direct patient care and interaction with a multidisciplinary team; (2) the experience in a third world country humbled them and made them appreciative of the resources available in the United States; and (3) weakness in the spacing of various assignments throughout the course.	No limitations mentioned

**Poirieret al. (2009) [61]**	Describes and evaluates the course developing cultural competency in pharmacy students	77 No demographics provided	Southern Illinois University Edwardsville, “*Health Promotion and Literacy*” Required course for third professional year	25-item pre- and post-assessment using IAPCC-R [137] on culture.	Scores improved on all constructs from pre- to post-, including awareness (14.5 to 16.5), knowledge (11.5 to 14.7), skill (11.8 to 15.7), encounters (12.9 to 15.1), desire (16.7 to 17.8) and the overall competence score (67.2 to 80.2, all *p* < 0.001). Course evaluation items ranged from 3.0 to 4.8 of six, with the highest items on development of knowledge on sociocultural groups and health literacy.	

**Prescott et al. (2019) [89]**	Assess first-year pharmacy students’ grades and preferred teaching styles within a multimodal teaching approach for developing cultural competency during a required Pharmaceutical Care I course.	136 57% women; 49% white, 38% Asian; mean age 21 years; 93% New York residents; 3% non-citizens	University at Buffalo School of Pharmacy Lecture and practicum within the first-year required Pharmaceutical Care I course focused on cultural knowledge and competency, including videos, structured discussion, and counseling sessions	Investigator-designed 9-item multiple choice knowledge assessment, rubric-graded assessment of counseling interactions, and 7-item reflection on themselves and their patients. Also included an 11-item survey on perceptions of the activity and their learning	For the knowledge assessment, students achieved a mean of 86.1% and median of 88.9%, with at least 85% on questions regarding national standards for CLAS with respect to healthcare, general family cultural differences, and some key terms (refugee, immigrant, integration, assimilation). Mean score on counseling was 92.6% and median was 94.7%. Among perceptions, students agreed that patient beliefs and cultural background are necessary for care (mean 4.7), patients’ personal goals are most important (3.9), they can communicate effectively with diverse patients (4.0), they have had culturally diverse relationships in their life (3.6), and they are culturally aware of people around them (4.1)	Quiz focused on facts; variability in teaching assistant grading; not designed to assess students’ previous cultural experiences; generalizability; focus specific to ethnicity, gender, and religion; lack of long-term data

**Saleset al. (2013) [62]**	Determine the degree to which three different educational interventions enhance cultural competency in pharmacy students	84 (26 lecture, 30 case-scenario, 28 simulation) 57% men; 81% white; 93% aged 20–25; 100% second professional year	University of Pittsburgh, “*Profession of Pharmacy 4 (POP4)*” Six-semester course series that provides exposure to various aspects of healthcare and pharmacy practice, one segment focuses on issues and challenges affecting the public’s health	15-item cultural assessment survey comprised of previously validated questions (from [151]) and original questions developed by the investigators. Three items on demographics and 12 items assessing six components of cultural competency (i.e., cultural awareness, cultural knowledge, cultural skills, cultural encounters, cultural desire, and cultural empathy) on a five-point Likert scale.	Overall changes from pre- to post- included enhanced agreement on items for ‘modify one’s interview when encountering diverse populations,’ (skills) and the statement ‘Patients prefer healthcare providers who are genuinely concerned with their cultural preferences’ (empathy). Students in the simulation (*p* = 0.008) and lecture (*p* = 0.037) groups showed greater improvement than those in the case-scenario group for the former.	Time length of interventions; insufficient follow-up time; sample size; not all students performed interview; response bias; lack of incentive

**Schweiger-Whalen et al. (2019) [86]**	(1) implement a pilot education program and test its effectiveness on the development of LGBT cultural competence in health care providers, and (2) evaluate the effect of a 4-hour pilot workshop on the development of LGBT cultural competence measured by quantitative tests and open-ended participant response and self-reflection	130 78.5% women; 53.1% white, 31.5% 20–29 years, 29.2% 30–39 years, 12.9% 40–49 years; 36.9% Hispanic/Latino; 57.7% nursing students; 83.8% heterosexual, 10% lesbian, gay or homosexual, 3.8% bisexual	New Mexico State University, *“Converging Cultures”* 4-hour pilot workshop (including lectures, panel discussion, and time for questions/answers) on the development of cultural competence offered at a regional community hospital and a local community college	Pre- and post-assessment using the 30-item GAP [119] on beliefs and behaviors regarding treatment of gay/lesbian patients, alongside an investigator-designed 13-item multiple choice knowledge quiz and open-ended questions on experience.	GAP results for nursing students included pre-workshop of 66.20 ± 6.35 increasing to 70.4 ± 6.64 post-workshop. Knowledge results for nursing students were 6.78 ± 2.21 and 10.09 ± 2.47 from pre- to post-workshop.	No control group; heterogenous sample; influence of politics and cultural beliefs in a single geographical area; generalizability; limited inclusion of racial minority groups

**Scisney-Matlock (2000) [63]**	Describe the effect of a target intervention derived from synthesizing multiple theories (social, cognitive, moral and ethical) and learning experiences on students’ perception of global diversity	120 (55 experimental and 65 control) Experimental: mean age 23.95 years; 91.2% women; 78% white. Control: mean age 24.35 years; 87% women; 80% white	University of Michigan Ann Arbor, “*Societal Health Issues*” Required undergraduate course	MLSS (from [62]) with five variables selected: diversity knowledge gained, knowledge of university diversity mandate, satisfaction with relevance of my ethnic group, reading of diversity, interaction with diversity. Data collected pre- and post- for experimental group, and post- only for control group.	Experimental group had stronger association for overall diversity knowledge compared to control (Adj R^2^ = 43.2% vs. 2%).	Generalizability; limited results

**Seymour et al. (2013) [64]**	Provide students with a systematic approach to ethical volunteering, critically reflecting on their motivation and attitudes related to conventional models of volunteering and facilitating alignment with principles of global health	30 No demographics provided	Harvard School of Dental Medicine, “*Workshop for Ethical Volunteering in Global Health*” Discussion-based interactive program required for third-year students	Investigator-designed pre-post survey rating importance of nine components of volunteering on a five-point Likert scale. Three components were conventional/traditional components aligned with voluntourism, two were convention but could be incorporated appropriately, and four were new standards for ethical volunteering.	Mean scores decreased for five of nine conventional/traditionally components post-, indicating decreasing importance. Scores for the four new standards increased post-, including “Working with the local government” (+.5%), “Participating long term (more than a few weeks)” (+7.3%), “Volunteering with a well-established organization” (+8.1%) and “Learning all about the community, including local customs and culture” (+17.5%).	Small sample size; failure to measure actual behaviors from volunteer experiences

**Smith and Silk (2011) [65]**	Investigate the impact of an online, interactive simulation involving an Arab American Muslim patient on the knowledge, skills, and attitudes of second-year medical students regarding culturally competent healthcare, both in general and specific to Arab American Muslim patients	199 (97 experimental and 102 control) 49.8% women; 78.3% Caucasian, 12.1% Asian American; 52.3% bilingual	Michigan State University College of Osteopathic Medicine, “*Behavioral System 1*” second-year course that included readings, small group discussions, a video, and a panel related to culturally competent healthcare	Investigators used the CCCQ. In addition, general diversity knowledge was with a seven-item scale composed, specific knowledge of Arab American Muslims measured with nine-item index, comfort level with patients from diverse backgrounds was measured with a nine-item scale and general cultural sensitivity was measured with a 12-item scale. Control group directly sent post-test measures, experimental group directed to an approximately 30- to 60-minute online, interactive patient simulation.	Main effect for condition on knowledge about Arab Americans (F [1, 195] = 4.85; p = 0.029), and perceived cultural sensitivity skill level with Arab Americans F [1, 195] = 7.04; p = 0.009. The online group reported more knowledge (MTreatment = 24.86; MControl = 22.19) about Arab Americans and greater self-efficacy (MTreatment = 2.80; MControl = 2.45) in communication. Additional main effect for bilingual status for five outcome measures, including knowledge of diversity (*p* = 0.001), knowledge (*p* = 0.001), overall cultural sensitivity (*p* = 0.036), cultural sensitivity skill level (*p* = 0.003), and cross-cultural comfort (*p* = 0.028).	Need or longer-term assessment; self-reported measure; generalizability to female students; selection bias; subjective assessment

**Smothers et al. (2019) [88]**	Determine if an educational intervention focused on the role of spirituality in healthcare positively affects medical students’ attitudes and perceptions relating to this topic	136 (84 intervention and 52 control) Intervention: 60% women; mean age 25 years; 48% white; 46% Christian, 26% Atheist Control: 56% women; mean age 26 years; 37% white; 49% Christian, 29% Atheist	Duke University School of Medicine 1-hour lecture and 1.5-hour small-group case-based discussion on religion/spirituality embedded within clinical skills foundation course for second-year medical students	Investigator-designed pre-post 18-item survey on attitudes, comfort, and perceptions using 10-point Likert scale items.	No differences in items pre-intervention, but differences in several items post-intervention, including appropriateness of a physician to share their own religious/spiritual beliefs with a patient (3.95 vs 4.76; p = 0.02), avoiding discussion of religion/spirituality with patients due to ethics (4.46 vs 3.85; p = 0.04), including religion/spirituality into care/practice (5.34 vs 6.59; p = 0.0013).	Reliance on self-reported data; selection bias; generalizability; non-validated tool

**Stiles et al. (2018) [81]**	Evaluate the effectiveness of a nursing stand-alone culture course with concurrent field experiences compared with the standard integrated culture content in the curriculum in increasing transcultural self-efficacy in nursing students.	72 (53 intervention and 19 control) Entire cohort: 13% men; 34.4% White, 18% Hispanic, 9.8% Black, 3.3% Native Indian/Hawaiian, 3.3% Asian, and 31.1% unknown;. 49.2% < 25 years, 44.3% 25–39 years, 7% > 40 years; 86% USA citizens, 8% non-USA born, 5% unknown	Sam Houston State University 15-week, two-credit nursing course, with 15 hours of lecture and 45 hours of clinical including faculty lectures about culture, student lectures on various cultural groups, and fieldwork/simulations with reflective journaling	Pre- and post-test design using the 83-item TSET [101].	Mean scores (pre- and post-intervention) for the cognitive subscale in the intervention group were 5.10 and 8.07, compared to 7.00 for the control group. Practical subscale scores were 5.56 and 8.27 for the intervention group, and 7.15 for controls. Affective subscale scores were 8.27 and 9.41 for intervention vs 8.85 for controls. On all subscales, there was a significant difference between pre- and post-assessments for treatment and control groups (p < 0.0001).	Unbalanced sample size in groups; lack of control over group assignment; some students assigned to a culture of study like their own; effect of demographic variables not widely explored; self-reported tool

**Strong et al. (2014) [66]**	Examined the effectiveness of an educational intervention designed to improve knowledge and attitudes of baccalaureate nursing students regarding LGBT patient care	58 6.9% first-year, 10.3% second-year, 34.5% third-year, 48.3% fourth-year; 10% heterosexual; 82.8% identify with a religion; 37.9% Democrats, 31% Republicans, 27.6% no political affiliation; 79.3% friend in LGBT community, 55.2% acquaintance, 24.1% family member; 56.9% reported positive or negative experience with the LGBT community	Illinois Wesleyan University 40–45 min educational presentation piloted with an expert panel from the university’s Pride Alliance	Three-item version (from [109]) of the Modified ATLG scale with five-point Likert scale for scoring, expanded to include items specific to bisexual and transgender individuals in a similar format. Six-item LGBT Healthcare scale, with three items based previous work from [152], and three additional items (perceptions of competence, cultural sensitivity skills, and nursing curricula) developed by the current research team. 15-item LGBT Knowledge questionnaire using T/F responses, with two items taken from the Knowledge About Homosexuality Questionnaire from [152] and 13 items developed by the current research team.	Mean results of the modified ATLG scale increased from pretest to posttest, specifically on lesbian, bisexual, and transgender (but not gay) subscales. Significant increases were seen for two items on the LGBT Healthcare scale, specific to competency to provide care and knowledge of specific health needs. A statistically significant increase in LGBT Knowledge questionnaire scores was noted (13.48 to 14.67, *p* < 0.001); five of 15 items were statistically significant between pretest and posttest.	No validated tools were found, so investigator-created tools were used; suboptimal reliability schedule of intervention affected depth; timing of recruitment impacted sample size; use of technology may have been a barrier; generalizability; cross-sectional data only

**Thompson et al. (2019) [94]**	Evaluate a gender-affirming healthcare curriculum for second-year medical students	129 47% women; 58.9% white, 19.4% Asian, 9.3% Hispanic, 5.4% African American/Black, 4.7% multiracial, 2.3% unknown	Rush Medical College Required gender-affirming healthcare curriculum with online videos, lecture slides, case-based learning, role-playing, practice questions, and panel discussions	Pre- and post-assessment using the gender identity adapted version of the SOPCS [113] including 26 items with 5-point Likert responses; two additional investigator items also assessed.	Total scale scores improved from pre-assessment (93.06 ± 10.12) to post-assessment (103.74 ± 11.94; p < 0.001), as well as skills (18.87 to 27.38) and knowledge (28.98 to 31.31), but not the attitudes subscale.	Single school; one profession

**Vela et al. (2008) [67]**	(1) Pilot a health disparities curriculum for incoming first year medical students and evaluate changes in knowledge; (2) Help medical students to become aware of personal biases regarding racial and ethnic minorities; (3) Inspire medical students to make a commitment to serve indigent populations	64 Approximately 50% women; 10% Hispanic, 5% African American; ~20% of students had received or knew someone who had received inferior care because of disparities in health; > 90% recalled reading or hearing about health disparities before the course	University of Chicago Pritzker School of Medicine, “*Health Care Disparities in America*” Intensive five-day elective course the week before orientation week offered to all matriculating students and incorporating the goals of the Society of General Internal Medicine (SGIM) Health Disparities Task Force	Investigator-designed pre-post surveys on factual knowledge (T/F, 13 items) and ratings (five-point Likert) of abilities to describe disparities and solutions (five items)	Students’ factual knowledge improved (76% to 89%, *p* < 0.0009) overall, and specifically on items “Physicians should attempt to ignore their own cultural background and biases when delivering health care to patients.” (32% to 53%, *p* = 0.02), “The Tuskegee Experiment was conducted to treat adult African Americans suffering from syphilis” (60% to 80%, *p* = 0.01), “About 50% of adult US citizens have deficient reading skills (functionally illiterate or have marginal reading skills).” (61 to 100%, *p* < 0.0001), “Young children within a non-English patient’s family should be used as interpreters as they are an excellent resource to diminish language barriers.” (74% to 98%, *p* = 0.001) and “Patient satisfaction tends to be higher if the patient and provider come from the same racial or ethnic background” (85% to 97%, *p* = 0.02). Ratings on all five description items also improved (all *p* < 0.001).	Self-selection; lack of validation; influence of pre-post repetition; lack of long-term follow-up

**Walton (2011) [68]**	Assess if there was a significant difference in cultural knowledge and awareness in college health science students before and after receiving education about Native Americans receiving hemodialysis	95 68.4% college students, 31.6% conference attendees; primarily Caucasian; age range 18–45 years; 79% participants aged 22 years and women	Liberal arts Catholic college in the rural Northwest (health science course) and Montana State Student Nurses Association conference 60-min educational presentation based on the research findings from “Prayer Warriors: A Grounded Theory Study of American Indians Receiving Hemodialysis”	18-item instrument on cultural awareness (attitudes, beliefs) based on previous research (from [153]) using three-point scale (most likely, neutral, not likely). Reflective papers post-presentations were analyzed using thematic analysis	Statistically significant improvements were noted for seven of 18 items. Qualitative themes included (1) approaching the patient with an open mind; (2) assessing beliefs and culture; (3) educating and re-educating the patient and family; (4) convincing the patient to begin dialysis; and (5) creating a sacred space.	Convenience sample; limited diversity; tool not tested for psychometric properties

**Williams et al. (2018) [72]**	Examine a set of self-perceptions defined as personal growth, attributed by graduate nursing students to an eight-week diversity course	41 90% women; mean age 34.3 years; 89% white, 8% African American, 3% other	University of Virginia School of Nursing, *“Culture and Health”* Required two-credit, 8-week summer diversity course including topics such as care access, social justice, and cultural competency for graduate nursing students	21-item PTGI [112] post-course assessment measuring positive psychological changes to coping in the wake of negative life experiences using a six-point Likert scale; students also completed a table of percentage attributions of cause of total personal growth	Mean level of total personal growth was 52.66 ± 21.65 and equivalent per-item mean was 2.51 ± 1.03 (between small and moderate total growth). Qualitative descriptions included “my clinical experiences,” “family challenges,” “interactions with diverse patients,” “met someone new,” “relationship with my patients,” and “getting married.”	High percentage of unusable surveys; reliance on introspective self-reported data; lack of pre-course data

**Yang et al. (2014) [69]**	Evaluate the effectiveness of a poverty simulation in increasing understanding of and attitudes toward poverty and resulting in changes in clinical practice among nursing seniors	199 No demographics provided	University of Pittsburgh Senior-level community health nursing course	Semi-structured five-item Poverty Simulation Reaction questionnaire (from [154]) using five-point Likert-type items. 21-item ATP scale (from [114])	Students significantly increased understanding of the financial pressures (2.89 ± 0.84 vs. 4.15 ± 0.58), difficult choices faced by individuals with few resources (2.76 ± 0.91 vs. 4.15 ± 0.68), challenges in improving situation (2.75 ± 0.88 vs. 4.10 ± 0.66), emotional stresses (2.89 ± 0.99 vs. 4.24 ± 0.70), and impact of social service system (2.59 ± 0.83 vs. 3.99 ± 0.77; all *p* < 0.001). 71% reported that the better understanding the lives of people in poverty. Most frequently reported feelings were frustration, stress, worthlessness, anxiety, and helplessness. After, students significantly changed in a positive direction in general (72.6 ± 9.3 vs. 75.9 ± 8.8; *p* = 0.001). and regarding feelings about stigma (24.9 ± 5.2 vs. 27.0 ± 4.8; *p* < 0.001). Qualitative data detailed an increased understanding of patients in poverty, an understanding of barriers to healthcare, increased empathy and influences on clinical practice.	Delivery of different assessments to different cohorts; differences in interactions during each simulation; role-playing may not simulate reality completely

**Yu et al. (2020) [74]**	Evaluate the feasibility and preliminary outcomes of immersive integrated experiential and didactic courses in strengthening competency-based global health learning in dental education	32 (didactic) and 3 (experiential) No demographics provided	Harvard School of Dental Medicine, *“Global Oral Health: Interdisciplinary Approaches”* and *“Global Health Extension Course: Perspectives from Costa Rica”* Required didactic and selective experiential competency-based global health courses for second-year dental students	Investigator-designed electronic survey on self-report of knowledge using a five-point Likert scale administered pre-course, immediately post-course, and at six months post-course; semi-structured interviews pre- and post-experiential course with another survey about the effectiveness of the experience.	Scores for different knowledge areas ranged from 2–3 pre-course to 3–4 immediately post-course, largely sustaining six-months post course. Themes from experiential student interviews focused on expert knowledge, discussion, field experience and comparisons to home healthcare system for learning modalities, and cultural competence, social determinants of health and local healthcare system for learned content.	Small sample size; bias in reporting learning gains; differences may reflect students intrinsically more interested in global health; one school

**Ziemba et al. (2016) [70]**	Document the challenges and rewards of the first pilot year of international videoconferencing in providing a low-cost, face to-face, real-time intercultural learning experience in community health nursing for undergraduate nursing students in two different countries, to discuss lessons learned, and provide recommendations for future semesters	22 (14 Haiti and eight USA) No demographics provided	University of Michigan and Faculty of Nursing Science of the Episcopal University of Haiti Videoconference activity part of one of nine clinical sections in the four-month semester in community health nursing	Formative and summative evaluations of written assignments/discussion, student evaluations of the experience.	Important things learned from peers included examples of information about their individual community assessments, opinions, and comparisons of the two countries and cultures. Challenges to communication included language, speed, earlier correspondence. Suggestions for improvement included goals, more course media, conversation in both languages.	Connection of data to actual learning objectives, lack of cultural competence measures


ATLG = Attitudes Toward Lesbians and Gay Men; ATN-SBIRT = Addiction Training for Nurses using Screening, Brief Intervention and Referral to Treatment; CAS = Cultural Assessment Survey; CCA = Cultural Competence Assessment; CCCHS = Caffrey Cultural Competence in Healthcare Scale; CCCAN-CLO = Culturally Congruent Care for Advanced Nursing Course Learning Objective; CCCQ = Clinical Cultural Competency Questionnaire; CCTDI = California Critical Thinking Dispositions Inventory; CISL = cultural immersion service learning; CLAS = Culturally and Linguistically Appropriate Services; CME = continuing medical education; CRI = critical reflective inquiry; CSI = Current Student Inventory; DSPS = Diverse Standardized Patient Simulation; ESI = Ethnic Sensitive Inventory; FORS = Faculty Observer Rating Scale; HBAS = Health Beliefs Attitudes Survey; HIV/AIDS = human immunodeficiency virus/acquired immunodeficiency syndrome; IAPCC = Inventory for Assessing the Process of Cultural Competence Among Healthcare Professionals; IAT = Implicit Association Test; IES = International Education Survey; IESS = Interprofessional Education Series Survey; IPEC = Interprofessional Education Collaborative; LEARN = listen, explain, acknowledge, recommend, negotiate; LGBTQIA = lesbian, gay, bisexual, transgender, questioning, intersex, and asexual; MAQ = Multicultural Assessment Questionnaire; MLSS = Michigan Longitudinal Study Scales; OSCE = Objective Structured Clinical Examination; OT = occupational therapy; PA = physician assistant; PMHNP = psychiatric-mental health nurse practitioner; PT = physical therapy; PTGI = Posttraumatic Growth Inventory; RIPLS = Readiness for Interpersonal Learning Scale; SAQ = Student Attitude Questionnaire; SOPCS = Sexual Orientation Provider Competency Scale; SOLER = square, open, lean, eye contact, relax; SP = standardized patient; TSET = Transcultural Self Efficacy Tool.

The studies include a broad array of literature with significant diversity (***Table 2***). The majority of the included articles assessed nursing education (38; 48.1%) [21,25,26,28,29,33,35,37,39,42,44,47,51,52,53,54,57,58,63,66,68,69,70,71,72,73,75,77,80,81,82,83,84,85,86,87,91,98], followed by pharmacy (26; 32.9%) [20,21,22,23,24,25,26,27,28,31,36,38,42,43,51,59,60,61,62] and medicine (25; 31.6%) [25,26,30,32,34,40,41,42,46,48,49,50,55,56,65,67,76,77,78,79,88,90,94,98]. A total of 12 studies described assessment of more than one health profession [21,25,26,28,31,34,42,51,54,68,77,98]. Cultural competency (56; 70.9%) [21,22,23,24,25,28,30,32,34,36,37,38,39,42,43,44,45,46,47,50,51,52,54,55,56,57,58,61,62,65,66,68,71,72,73,75,77,78,79,80,81,82,83,84,85,87,88,89,90,91,93,94,95,96,97,98] was the most common aspect of global health subject area, and most studies reported data resulting from standard live didactic course structures (58; 73.4%) [20,22,23,24,25,26,28,29,30,31,32,36,38,39,40,41,42,44,45,46,47,48,49,51,52,54,55,56,57,59,61,62,63,64,65,67,69,70,71,72,73,74,75,76,80,81,82,83,84,85,86,87,88,89,95,96,97,98] A variety of methodologies were utilized to measure outcomes, but the most common were survey questionnaires (68; 86.1%) [20,21,22,23,24,25,26,27,28,29,30,31,32,33,34,35,36,37,38,39,40,41,42,44,46,47,49,50,51,52,55,57,59,60,61,62,63,64,65,66,67,68,69,71,72,73,74,75,76,77,78,79,81,83,85,86,88,89,90,91,92,93,94,95,96,97,98] which were administered before and after educational interventions (51; 64.6%) [20,21,22,23,24,25,26,27,28,29,31,32,35,36,37,38,40,42,44,46,49,50,51,52,57,60,61,62,63,64,65,66,67,68,69,71,74,75,76,77,78,85,86,88,91,92,93,94,95,96,97] primarily collecting quantitative data (68; 86.1%) [20,21,22,23,24,25,26,27,28,29,30,31,32,33,34,35,36,37,38,39,40,41,42,44,46,47,49,50,51,52,55,57,59,60,61,62,63,64,65,66,67,68,69,71,72,73,74,75,76,77,78,79,81,83,85,86,87,88,89,90,91,92,93,94,95,96,97,98]. Each study measured some aspects of students’ attitudes, perceptions, and beliefs regarding their educational outcomes. Fewer than half of the studies utilized objective knowledge assessments (34; 43.0%) [20,22,25,28,31,32,35,36,37,38,40,42,43,44,46,47,49,50,51,57,61,62,65,66,67,71,74,79,89,92,94,95,96,97]. Finally, sample sizes were split relatively equally among small (26; 32.9%) [25,27,31,32,33,37,38,40,43,48,49,52,53,56,57,58,60,64,70,72,74,82,83,87,91,95], medium (30; 38.0%) [20,21,23,28,34,36,44,45,50,59,61,62,66,67,68,71,73,76,77,80,81,84,85,86,88,90,92,94,96,97] and large studies (23; 29.1%) [22,24,26,29,30,35,39,41,42,46,47,51,54,55,63,65,69,75,78,79,89,93,98].

**Table 2:** Description of the Study Characteristics.


CHARACTERISTIC	N (%)	REFERENCES

Student learners assessed^a^		

Nursing/midwifery	38 (48.1)	[21,25,26,28,29,33,35,37,39,42,44,47,51,52,53,54,57,58,63,66,68,69,70,71,72,73,75,77,80,81,82,83,84,85,86,87,91,98]

Pharmacy	26 (32.9)	[20,21,22,23,24,25,26,27,28,31,36,38,42,43,51,59,60,61,62,77,89,92,93,95,96,97]

Medicine	25 (31.6)	[25,26,30,32,34,40,41,42,46,48,49,50,54,55,56,65,67,76,77,78,79,88,90,94,98]

Dentistry	5 (3.6)	[25,26,45,64,74]

Physical therapy^b^	4 (5.1)	[25,34,42,98]

Social work^b^	4 (5.1)	[28,31,54,98]

Occupational therapy^b^	2 (2.5)	[42,54,98]

Public health^b^	1 (1.3)	[42,54,98]

Nutrition^b^	1 (1.3)	[21]

Topic area^a^		

Cultural competence	56 (70.9)	[21,22,23,24,25,28,30,32,34,36,37,38,39,42,43,44,45,46,47,50,51,52,54,55,56,57,58,61,62,65,66,68,71,72,73,75,77,78,79,80,81,82,83,84,85,87,88,89,90,91,93,94,95,96,97,98]

Disparities	21 (26.6)	[25,26,27,29,31,35,40,41,46,47,48,49,59,67,69,77,86,87,90,92,96]

Global health	10 (12.7)	[20,33,53,60,63,64,70,74,76,90]

Type of course^a^		

Live didactic course	58 (73.4)	[20,22,23,24,25,26,27,28,29,30,31,32,36,38,39,40,41,42,44,45,46,47,48,49,51,52,54,55,56,57,59,61,62,63,64,65,67,69,70,71,72,73,74,75,76,80,81,82,83,84,85,86,87,88,89,95,96,97,98]

Service-learning course	23 (29.1)	[21,27,31,32,33,34,35,37,43,48,53,54,57,58,60,65,73,74,77,78,82,83,90]

Online didactic course	8 (10.1)	[20,30,32,44,57,79,83,91]

Live presentation	6 (7.6)	[50,66,68,92,93,94]

Study methodology utilized^a^		

Quantitative	68 (86.1)	[20,21,22,23,24,25,26,27,28,29,30,31,32,33,34,35,36,37,38,39,40,41,42,44,46,47,49,50,51,52,55,57,59,60,61,62,63,64,65,66,67,68,69,71,72,73,74,75,76,77,78,79,81,83,85,86,87,88,89,90,91,92,93,94,95,96,97,98]

Survey questionnaire	68 (86.1)	[20,21,22,23,24,25,26,27,28,29,30,31,32,33,34,35,36,37,38,39,40,41,42,44,46,47,49,50,51,52,55,57,59,60,61,62,63,64,65,66,67,68,69,71,72,73,74,75,76,77,78,79,81,83,85,86,87,88,89,90,91,92,93,94,95,96,97,98]

Pre/post assessment	51 (64.6)	[20,21,22,23,24,25,26,27,28,29,29,31,32,35,36,37,38,40,42,44,46,49,50,51,52,57,60,61,62,63,64,65,66,67,68,69,71,74,75,76,77,78,85,86,88,91,92,93,94,95,96,97]

Quasi-experimental	8 (10.1)	[22,23,52,62,63,81,86,94]

Randomized	2 (2.5)	[30,65]

Qualitative	24 (30.3)	[33,37,43,45,47,48,53,54,56,58,59,68,69,70,71,73,74,76,77,80,82,84,90,92]

Outcomes assessed^a^		

Attitudes/perceptions/beliefs	72 (91.1)	[20,21,22,23,24,25,26,27,28,29,30,31,32,33,34,35,36,37,38,39,40,41,42,43,44,45,46,47,48,49,50,51,52,53,54,55,56,57,58,59,60,61,62,63,64,65,66,67,68,69,70,71,72,73,75,77,79,80,81,82,83,84,85,86,87,88,89,90,91,92,93,94,95,96,97,98]

Knowledge	34 (43.0)	[20,22,25,28,31,32,35,36,37,38,40,42,43,44,46,47,49,50,51,57,61,62,65,66,67,71,74,79,87,89,92,94,95,96,97]

Skills	15 (19.0)	[28,31,32,37,38,42,44,51,57,61,62,65,78,79,94]

Study sample size		

Less than 50	26 (32.9)	[25,27,31,32,33,37,38,40,43,48,49,52,53,56,57,58,60,64,70,72,74,82,83,87,91,95]

51–100	30 (38.0)	[20,21,23,28,34,36,44,45,50,59,61,62,66,67,68,71,73,76,77,80,81,84,85,86,88,90,92,94,96,97]

More than 100	23 (29.1)	[22,24,26,29,30,35,39,41,42,46,47,51,54,55,63,65,69,75,78,79,89,93,98]

^a^ Studies may satisfy more than one characteristic. ^b^ Inclusion criteria for the review only specifically searched for studies regarding health professional students in medicine (including osteopathy and physician assistants), dentistry, nursing (including midwifery) and pharmacy. Other health professional students may have been included in studies that met the aforementioned criteria but were not specifically searched and cannot be considered comprehensive.

A breakdown and crosstabulation of individual assessment methods within included articles is summarized in ***Table 3***. Details regarding the constructs of validated tools are provided in ***Table 4***.

**Table 3:** Description of Educational Measurement Strategies for Global Health Courses.


	CULTURAL COMPETENCE		HEALTH DISPARITIES		GLOBAL HEALTH	
		
	*QUANTITATIVE*	QUALITATIVE*	*QUANTITATIVE*	QUALITATIVE*	*QUANTITATIVE*	QUALITATIVE*

**Attitudes/Perceptions/Beliefs**	Validated: ATLG [66] CAS [71] CCA [75,91] CCCQ [51,65] CCCHS [21,77] HBAS [30] IAPCC [28,37,38,42,44,57,61,73] IDI [83] IESS [34] MAQ [32] PTGI [72] RIPLS [77] SOPCS [94] TSET [52,81,85] UCSF Cultural Competence Tool [24] Not validated: [22,25,28,36,39,46,47,50,52,55,62,66,68,79,88,89,90,91,93,95,96,97,98]	Alexander-Ruff andKinion [82] (instructor observations & student reflections, constant comparative method) Chang et al. [90] (journal entries, grounded theory) Doutrich and Storey [37](recorded workshops & reflective essays, thematic analysis & phenomenology) Heffernan et al. [43] (focus groups, thematic analysis) Kamau-Small et al. [47](short answer reflections & clinical application reports, Stages of Change & TBF) Knecht et al. [73] (focus group, content analysis) Isaac etal. [45] (reflective essay, LIWC & factor analysis) Matsunaga et al. [54] (reflective essays, grounded theory) McElfish [77] (focus groups, thematic analysis) Miller and Green [56] (semi-structured interviews, thematic analysis) Mkanawire-Valhmu et al. [84] (reaction papers, thematic analysis) Olukotun etal. [80] (reflective essays, Krathworth taxonomy) Ott et al. [58](debriefings & focus groups & papers, thematic analysis) Walton [68] (reflective essay, thematic analysis)	Validated: ATP [69] Centers for Healthy Communities Survey [26,35,56] ESI [87] GAP [86] IAT [41] Transphobia Scale [25] Not validated: [26,27,29,31,40,41,46,47,49,59,67,69,79,90,92,96]	Chang et al. [90] (journal entries, grounded theory) Kamau-Small et al. [47] (short answer reflections & clinical application reports, Stages of Change & TBF) Knockel et al. [92] (short answer reflections, content analysis) Lee et al. [48] (reflective essays, thematic analysis) McElfish [77] (focus groups, thematic analysis) Parkhill et al. [59] (reflective essays, thematic analysis) Yang etal. [69] (short answer reflections, thematic analysis)	Validated: IES [33] MLSS [63] Not validated: [20,60,64,76,90]	Chang etal. [90] (journal entries, grounded theory) Curtin et al. [33](reflective essays, critical reflection inquiry & content analysis) Main et al. [53] (reflective journals, content analysis) Peluso et al. [76] (shortanswer reflections, grounded theory) Yu et al. [74] (semi-structuredinterviews and reflection essays, content analysis) Ziemba et al. [70](short answer reflections, frequencies of concepts)

**Knowledge**	Validated: CCCQ [51,65] IAPCC [28,37,38,42,44,57,61,73] MAQ [32] SOPCS [94] Not validated: [22,25,36,46,50,51,62,66,79,89,95,96,97] Grading: [47]	Heffernan et al. [43] (focus groups, thematic analysis)	Validated: Centers for Healthy Communities Survey [26,35,56] Not validated: [25,31,40,46,49,67,79,92,96] Grading: [47]		Not validated: [20,74] Grading: [20,60]	

**Skills**	Validated: CCCQ [51,65] FORS [78] IAPCC [28,37,38,42,44,57,61,73] MAQ [32] SOPCS 94] Not validated: [62,78,79]		Not validated: [31,79]			


ATLG = Attitudes Toward Lesbians and Gay Men; ATP = Attitude Toward Poverty; CAS = Cultural Assessment Survey; CCA = Cultural Competence Assessment; CCCHS = Caffrey Cultural Competence in Healthcare Scale; CCCQ = Clinical Cultural Competency Questionnaire; ESI = Ethnic Sensitive Inventory; FORS = Faculty Observer Rating Scale; GAP = Gay Affirmative Practice; HBAS = Health Beliefs Attitudes Survey; IAPCC = Inventory for Assessing the Process for Cultural Competency; IAT = Implicit Association Test; IDI = Intercultural Development Inventory; IES = International Education Survey; IESS = Interprofessional Education Series Survey; Linguistic Inquiry and Word Count = LIWC; MAQ = Multicultural Assessment Questionnaire; MLSS = Michigan Longitudinal Study Scales; PTGI = Post-traumatic Growth Inventory; RIPLS = Readiness for Interprofessional Learning; SOPCS = Sexual Orientation Provider Competency Scale; TBF = Transfer Barriers Framework; TSET = Transcultural Self-Efficacy Tool. * Qualitative description includes the data source and analytic strategy.

**Table 4:** Description of Global Health Assessment Tools.


ASSESSMENT TOOL NAME	SUBJECT AREA	ITEMS (N)	CONSTRUCTS MEASURED	REFERENCES

Attitudes Toward Lesbians and Gay Men (ATLG)	CC	3–10	Attitudes, homophobia	[66]

Attitude Toward Poverty (ATP/ATP-SF)	HD	21	Student attitude toward people living in poverty	[69]

Cultural Assessment Survey (CAS)	CC	17	Awareness of cultural diversity and biases regarding cultural issues/practices	[71]

Cultural Competence Assessment (CCA)	CC	44	Cultural awareness and sensitivity, cultural competence behavior	[75,91]

Caffrey Cultural Competency in Healthcare Scale (CCCHS)	CC	28	Perceived knowledge, comfort (proximal/distal), awareness	[21,77]

Centers for Healthy Communities Survey	HD	15	Civic engagement, cultural competence, social issues, health disparities	[26,35,56]

Clinical Cultural Competency Questionnaire (CCCQ)	CC	86	Knowledge, skills, comfort with encounters/situations, attitudes, education, and training	[51,65]

Ethnic Sensitive Inventory (ESI)	HD	24	Sensitivity among mental health professionals during treatment phases (precontact, problem identification, problem specification, mutual goal formulation, problem solving, and termination)	[87]

Faculty Observer Rating Scale (FORS)	CC	11	Observation of skills related to working with an interpreter in a patient interview	[78]

Gay Affirmative Practice (GAP)	HD	30	Beliefs and behaviors regarding treatment of gay/lesbian patients	[86]

Health Beliefs Attitude Survey (HBAS)	CC	15	Confidence to assess patient perspectives and opinions, beliefs, psychosocial and cultural context during medical interviews	[30]

Implicit Association Test (IAT)	HD	N/A	Detect the strength of person’s subconscious associations	[41]

Intercultural Development Inventory (IDI)	CC	50	Perceived and actual intercultural competence as defined on a continuum	[83]

International Education Survey (IES)	GH	29	Professional nurse role dimension, international perspectives, personal development, intellectual development	[33]

Interprofessional Education Series Survey (IESS)	CC	10	Engagement and attitudes towards interprofessional education	[34]

Inventory for Assessing the Process of Cultural Competence Among Healthcare Professionals-Revised/Student Version (IAPCC-R/SV)	CC	20	Desire, awareness, knowledge, skill and encounters	[28,37,38,42,44,57,61,73]

Michigan Longitudinal Study Scale (MLSS)	GH	N/A	Diversity knowledge gained, knowledge of university diversity mandate, satisfaction with relevance of my ethnic group, reading of diversity, interaction with diversity	[63]

Multicultural Assessment Questionnaire (MAQ)	CC	16	Knowledge, skills, and attitudes	[32]

Post-traumatic Growth Inventory (PTGI)	CC	21	Endorsement of positive psychological changes as to the degree to which they apply to the respondent	[72]

Sexual Orientation Provider Competency Scale (SOPCS)	CC	26	Skills, attitudes, and knowledge regarding gender identify competency	[94]

Transcultural Self-Efficacy (TSET)	CC	83	Confidence in cognitive (knowledge), practical (interview), or affective (attitudes, values, and beliefs) skills	[52,85]

Transphobia Scale	HD	9	Gender bashing, genderism, transphobia	[25]

UCSF cultural competence tool	CC	12	Awareness, knowledge, communication skills, cross-cultural communication	[23,24]


CC = Cultural competence; GH = global/international health; HD = health disparities; UCSF = University of California, San Francisco.

### Quantitative assessment methods

#### Validated quantitative tools

For cultural competence, the most commonly used validated tool was the Inventory for Assessing the Process of Cultural Competence (IAPCC), with different versions developed by Campinha-Bacote et al. [99,100], was used in eight studies [28,37,38,42,44,57,61,73,81] The following validated tools were used at least once: the Transcultural Self Efficacy Tool (TSET) [101], the Clinical Cultural Competency Questionnaire (CCCQ) [52,85,102] the Cultural Competence Assessment (CCA) [75,91,103], the Multicultural Assessment Questionnaire (MAQ) [32,104] the Caffrey Cultural Competence in Healthcare Scale (CCCHS) [21,77,105], the Cultural Assessment Survey (CAS) [71,106] the Health Beliefs Attitude Survey (HBAS) [30,107,108], the Attitudes Toward Lesbians and Gay Men (ATLG) [66,109] the Faculty Observer Rating Scale (FORS) [78,110], the Intercultural Development Inventory (IDI) [83,111], the Posttraumatic Growth Inventory (PTGI) [72,112], the Sexual Orientation Provider Competence Scale (SOPCS) [94,113], the UCSF cultural competence and cross-cultural communication tool created and validated by Assemi and colleagues [23,24 ], and the Interprofessional Education Series Survey (IESS) [34].

Several tools in related domains were utilized in the subject areas of global health and health disparities. In health disparities, four validated tools were identified. Each tool was used by at least one study: the Attitude Toward Poverty (ATP) scale [69,114], the Ethnic Sensitive Inventory (ESI) [87,115] a transphobia scale by Braun and colleagues [25,116], the Implicit Association Test (IAT) [41,117,118], the Gay Affirmative Practice (GAP) scale [86,119], and the Centers for Healthy Communities Survey [26,35,56,83]. Two validated tools were used in the area of global health, the International Education Survey (IES) [33,120] and the Michigan Longitudinal Study Scales [63]. Although two articles used the California Critical Thinking Disposition Inventory (CCTDI) [37,57] and two articles used the Readiness for Interprofessional Learning Scale (RIPLS) [77,121], these tools were not included because they did not specifically measure a construct related to global health.

#### Non-validated quantitative tools

Several articles used investigator-designed quantitative assessment tools. These tools were most commonly used in the subject area of cultural competence (25) [22,25,28,36,39,46,47,50,51,52,55,62,66,68,78,79,88,89,90,91,93,95,96,97,98], followed by health disparities (17) [25,26,27,29,31,40,41,46,47,49,59,67,69,79,90,92,96], and global/international health (6) [20,60,64,74,76,90]. Some instruments were based on previously published but non-validated tools [22,28,36,39,46,47,62,66], while others described a process of ensuring face validity [25,36,62,88,91] Several studies also reported results of graded assignments as an assessment tool [19,46,59].

### Qualitative assessment methods

Several studies used qualitative methodologies to evaluate student learning, particularly focusing on attitudes, perceptions, and beliefs. Eleven studies used qualitative evaluations alone [43,45,48,53,54,56,58,70,80,82,84] while twelve studies used a combination of quantitative and qualitative methodologies to assess coursework [37,47,59,68,69,71,73,74,76,77,90,92].

Fourteen studies evaluated a course focusing on cultural competence used qualitative assessments. Researchers used a diverse group of data sources for their qualitative evaluations including reflective essays [37,45,47,54,68,80,82] reflective journals [54,90], focus groups [43,58,73,77,82] semi-structured interviews [56], student written assignments [48,58,84], and recorded workshops/debriefs [37,58], short answer reflections [47], and instructor observations [82]. The most common analytic approach was thematic analysis; [37,43,54,56,58,68,77,84] other studies employed the constant comparator method [82], grounded theory [54,90], or content analysis [73]. One analysis used interpretive phenomenology as a strategy [37], while another [45] used Linguistic Inquiry & Word Count (LIWC) and factor analysis to describe the content of student reflective essays. One study [47] applied the Transfer Barrier Framework [122] and Prochaska and DiClemente’s Stages of Change Model [123] to written narratives prior to analysis, while a second study [80] used Krathwohl’s Taxonomy [124] as a thematic framework for analysis of reflective essays.

Seven of the studies that evaluated health disparities used qualitative methodologies. Data sources for health disparities evaluations included reflective essays and journals, [47,48,59,90]short answer reflective responses, [47,69,92] student written assignments, and focus groups [47,77]. Four studies [48,59,69,77] conducted thematic analyses, one study used content analysis [92], while another employed grounded theory [90]. Six qualitative global health evaluations included reflective essays [33,74], reflective journals [53,90], short answer reflections [70,76], written assignments [33], and semi-structured interviews [74]. Most of the studies that described their analysis strategy used content analysis [33,53,74] or grounded theory [76,90].

## Discussion

This systematic review examined the diverse scope of assessment methods used in global health education for health professions students in the didactic setting. The results of this review expand the knowledge on global health in current literature. A recent scoping review by Costa Mendes et al. described the diversity, structure, models, emotional, cultural, and collaborative aspects of teaching global health in the Americas [125]. In this review, the authors recommended more consistency and standardization in educational approaches, echoing similar sentiments to a previous systematic review of global health graduate medical education by Bills and Ahn [88]. Despite these previous reviews providing excellent discussions on the educational strategy landscape, there have been limited publications specifically focused on assessment. The most relevant is a recent scoping review by Schleiff et al. [126], which examined literature on competency-based training incorporation into curricula and evaluation strategies, with a subset focused on clinical and allied health professional training. This review identified three major forms of evaluation tools, including self-assessment surveys, assessment from multiple stakeholders’ perspectives, and mixed-methods approaches. This current research corroborates these themes and provides an in-depth examination of specific assessment strategies.

This study used a broad lens of global health while allowing for more refine selection of assessment tools based on into three distinct topic areas. The largest category of tools focused on cultural competence that measured it broadly or cross-cultural care specifically among health professions students. Almost all these tools utilized self-reported perceptions or behaviors using a Likert-type format. Although one of these tools, the IAPCC-SV, was developed for undergraduate students, it was utilized by several graduate programs, including pharmacy, medicine, nursing, and dentistry. The advantage of this tool is its relatively short format compared with others; however, its cost may be a challenge for a wide adoption. The related IAPCC-R requires an advanced reading level for comprehension and was developed specifically for a nursing audience. This tool also requires a fee for use with a process to obtain permission for use, with the online version costing more than the paper version. In addition, the TSET, CCCQ, CCCHS. and CCA were each used in multiple studies. The TSET is validated for use in nursing students to measure student transcultural self-efficacy perceptions related to the performing of general transcultural nursing skills in a diverse client population [101]; the tool may be useful for pre/post learning evaluation and demonstrate changes over time. The CCCHS is designed to measure self-perceived knowledge, self-awareness, and comfort with skills of cultural competence [105]. This measure was used to assess nursing, pharmacy, and dental students with a strength being the ability to show student improvement over time [21,52,77,85]. The CCA was designed as a pre/post learning tool and measures perceptions of experiences with diverse groups, awareness, and sensitivity [103]. The strength of the CCA is the potential use in variety of healthcare provider populations of different educational levels. Although CCCQ and MAQ were originally developed for practicing physicians, they have been used by other healthcare professionals to assess knowledge, skills, and attitudes. In all measures described, there must be consideration for the level of objectivity due to the self-report nature of the tools and the fact that evidence is lacking that attitudes translate to behavior. The updated search highlighted a trend toward a greater emphasis in global health education reflected in the high number of included studies. Approximately one-third of the studies in this review, those that included validated tools, were published recently (2017–2020) [71,72,75,77,78,83,86,87,91,94]. While the updated search included more studies, the tools were primarily designed to assess cultural competence, there were a few that assessed health disparities parameters such the ESI and the GAP scales [86,87].

While diverse assessment tools were used to measure global health outcomes broadly, global health comprise more than cultural competency. Only eight studies included in this review specifically assessed global health-related outcomes, with only two using a validated tool. This work highlights the need for global health educators to develop and validate innovative strategies in assessment, particularly in the area of didactic global health education. Similar to these findings, Kiles et al. [127] further highlighted that there were gaps in the literature regarding global health education. While their review focused on teaching strategies rather than assessing educational outcomes, it also recognized the need for further research on teaching strategies and assessments that may be used to assess the complex skills needed in the global health setting including understanding organizational factors, global healthcare system, and SDOH.

In addition to validated survey tools, qualitative assessment methods may be used to assess cultural competency, health disparities and global health. Reflective essays, journal, focus groups, structured interviews and recorded debriefing sessions were used in this review, and most often evaluated student attitudes, perceptions, and beliefs. As with the quantitative assessments, cultural competency was the topic most frequently assessed. Although qualitative tools are useful for assessment, there were fewer qualitative studies included due the lack of rigor in the research methodologies of many of the studies. The use of qualitative tools to assess global health skills is an area for further investigation.

While the topic of cultural competency/sensitivity is important for enhancing the knowledge, skills, and attitudes of students, these are only one part of the whole patient and system of health care. New effective assessment strategies are needed for examining student knowledge of frameworks of holistic patient care, including the social ecological model [128]. Frameworks and theories that emphasize structural competency, including those described by Metzl and Hensen [129] could be considered when developing global health assessment tools.

Complex global health problems are better solved through teamwork. Experts note that interprofessional education may assist with preparing health care providers for the challenges of working in resource-limited settings [130]. This current review aimed to be inclusive of major health professions, in line with the understanding that students should be trained to solve complex problems in interprofessional teams with a focus on broader contextual factors that influence health [91]. However, a limited number of studies identified included multiple health professions in their assessments. Application of these assessment tools in interprofessional settings is needed in order to fully evaluate their usefulness. There are persistent and glaring inequities in health worldwide, despite decades of initiatives to promote social justice, disparities have become even more evident during the COVID-19 pandemic [92]. Highlighted are the structural problems that lead to disparities. Health professional education acknowledges the deficits in the current system [131]. Current assessment tools provide limited focus on the evaluation of the essential knowledge and skills needed by students to address the complex factors that affect the health of populations including the social determinants of health [127]. Moreover, because heath care professionals are confined by organizational, institutional, and governmental policies and regulations, an assessment of their cultural competency alone does not address the whole picture. The overall goal of global health education is rooted in health equity and social justice. It incorporates the multiple structural factors that impact individuals and populations. Structural competency is a framework that considers the knowledge, skills, and attitudes of the individual healthcare professionals while complementing awareness and action toward the structural changes needed in systems and policies [129]. However, it is noted that in studies that assess cultural competence alone, consensus was found to be complex [132]. An assessment of structural competency may only add to this complexity. Although acquiring skills is an essential element of global health competency, skills were measured least frequently and there is a lack of validated instruments to measure them. Thus, there is a critical need for a validated instrument designed to assess skills in global health among future healthcare professionals. Consequently, additional research is needed to address this gap.

Although this study has many strengths, it is important to acknowledge the study limitations. First, this review may have excluded relevant literature due to lack of quality. For instance, some articles were excluded because their qualitative assessments lacked descriptions of methodology. Second, because the field of global health is constantly evolving, the lack of standardized definitions and terminology in the included studies was challenging. In an effort to be inclusive, the authors broadly considered the global health constructs, including health disparities and cultural competency. However, despite this inclusivity in the root understanding of the definition of global health, the review did place limits on other aspects of the research objective, including which health professions were included, and focused solely on didactic education rather than including experiential evaluations. While there are frameworks and competencies, validated assessment tools will be easier to develop with consensus on a definition. Third, this study was conducted by faculty from schools/colleges of pharmacy. Global health is by design interprofessional, the inclusion of viewpoints from other health professions would enhance the interpretation of the results. Finally, the approach was to design the literature search method to be as comprehensive as possible, which resulted in a large result set that required significant time to evaluate. Accordingly, this review does not include studies from outside of the United States.

Despite these limitations, this study has several strengths. First, this research reviewed over two decades of literature on assessment methods in didactic education in global health. Second, this systematic review consisted of studies of learners from multiple healthcare professions increases the generalizability of the results. Third, this research categorized quantitative and qualitative data, assessments focusing on attitudes, perceptions, and beliefs as well as knowledge and skills, and validated and non-validated assessments. This comprehensive approach allows educators to consider the breadth and depth of available instruments to aid their own course assessment methods for what best fits their needs.

## Conclusion

This systematic review examined effective assessment strategies for didactic global health education. Specifically, this research provided a comprehensive list of validated tools to utilized to assess students’ competencies. Based on the findings of this study, there is a lack of adequate assessment tools for health disparities. Moreover, there is a need for consistent assessment strategies in didactic global health education. More research is needed in assessment to ascertain the complex attitudes and skills required for student competence in holistic patient-centered care. Given the recent pandemic with existing health inequities, health professionals must be equipped to be leaders in health equity and global health.
